# Information Quality Frameworks for Digital Health Technologies: Systematic Review

**DOI:** 10.2196/23479

**Published:** 2021-05-17

**Authors:** Kayode Philip Fadahunsi, Siobhan O'Connor, James Tosin Akinlua, Petra A Wark, Joseph Gallagher, Christopher Carroll, Josip Car, Azeem Majeed, John O'Donoghue

**Affiliations:** 1 Department of Public Health and Primary Care Imperial College London London United Kingdom; 2 School of Health in Social Science The University of Edinburgh Edinburgh United Kingdom; 3 Department of Primary Care and Population Health Sciences University College London London United Kingdom; 4 Centre for Intelligent Healthcare Institute of Health and Wellbeing Coventry University Coventry United Kingdom; 5 gHealth Research Group School of Medicine University College Dublin Dublin Ireland; 6 Health Economics and Decision Science School of Health and Related Research The University of Sheffield Sheffield United Kingdom; 7 Centre for Population Health Sciences LKC Medicine Nayang Technological University Sungapore Singapore; 8 Malawi eHealth Research Centre University College Cork Cork Ireland; 9 ASSERT Research Centre University College Cork Cork Ireland

**Keywords:** digital health, patient safety, information quality

## Abstract

**Background:**

Digital health technologies (DHTs) generate a large volume of information used in health care for administrative, educational, research, and clinical purposes. The clinical use of digital information for diagnostic, therapeutic, and prognostic purposes has multiple patient safety problems, some of which result from poor information quality (IQ).

**Objective:**

This systematic review aims to synthesize an IQ framework that could be used to evaluate the extent to which digital health information is fit for clinical purposes.

**Methods:**

The review was conducted according to the PRISMA (Preferred Reporting Items for Systematic Reviews and Meta-Analysis) guidelines. We searched Embase, MEDLINE, PubMed, CINAHL, Maternity and Infant Care, PsycINFO, Global Health, ProQuest Dissertations and Theses Global, Scopus, and HMIC (the Health Management Information Consortium) from inception until October 2019. Multidimensional IQ frameworks for assessing DHTs used in the clinical context by health care professionals were included. A thematic synthesis approach was used to synthesize the Clinical Information Quality (CLIQ) framework for digital health.

**Results:**

We identified 10 existing IQ frameworks from which we developed the CLIQ framework for digital health with 13 unique dimensions: accessibility, completeness, portability, security, timeliness, accuracy, interpretability, plausibility, provenance, relevance, conformance, consistency, and maintainability, which were categorized into 3 meaningful categories: availability, informativeness, and usability.

**Conclusions:**

This systematic review highlights the importance of the IQ of DHTs and its relevance to patient safety. The CLIQ framework for digital health will be useful in evaluating and conceptualizing IQ issues associated with digital health, thus forestalling potential patient safety problems.

**Trial Registration:**

PROSPERO International Prospective Register of Systematic Reviews CRD42018097142; https://www.crd.york.ac.uk/prospero/display_record.php?RecordID=97142

**International Registered Report Identifier (IRRID):**

RR2-10.1136/bmjopen-2018-024722

## Introduction

### Background

Digital health—the use of digital technologies for health—is increasingly recognized as a major driver of quality in health care [1]. Digital health technologies (DHTs), such as telemedicine, electronic health records (EHRs), clinical decision support systems (CDSS), mobile health, computerized physician order entry, electronic prescribing systems, and web-based health services, can improve access and quality of health care services [2,3]. DHTs generate a copious amount of information used in health care for administrative, educational, research, and clinical purposes [4,5]. However, the clinical use of digital information for diagnostic, therapeutic, and prognostic purposes has multiple patient safety problems, including significant harms and death, some of which result from poor information quality (IQ) [6-9]. For instance, a patient in the United Kingdom experienced a life-threatening allergic reaction following a medication error because of inaccessible allergy information in the EHR [6].

IQ refers to the extent to which information is fit for a specific purpose [10,11]. IQ is multidimensional, with each dimension describing a unique aspect of information [10,12]. For example, accuracy describes the extent to which information is correct, and accessibility describes the extent to which information is easily obtainable [12]. Dimensions relating to a specific context are traditionally integrated into a framework for evaluating IQ within the context [10,11]. One IQ framework for EHRs [13] has 11 dimensions and 3 categories, as shown in Textbox 1. The framework depicts the relationship between the dimensions by categorizing statistically measurable dimensions as objectivity, security-related dimensions as integrity, and dimensions relating to the usefulness of information to intended users as utility [13].

**Dimensions and categories in an information quality framework for electronic health record.**

Objectivity

- Accuracy
- Completeness
- Consistency
- Timeliness

Utility

- Provenance
- Interpretability
- Usability
- Relevance

Integrity

- Privacy
- Confidentiality
- Secure access

### Research Problem and Objective

Currently, there is no consensus on the definition of IQ dimensions in the context of the use of digital health information for clinical purposes. There is a lack of consistency in the terminology and definition of dimensions in existing IQ frameworks, limiting a common understanding of IQ requirements for DHTs [14]. Although previous literature reviews have attempted to define the IQ dimensions of digital health information, they focused on the use of digital health information for administrative and research purposes [14,15]. Identifying and defining IQ dimensions in the context of the use of digital health information for clinical purposes is especially important considering the patient safety implications of poor IQ, as discussed earlier [6-8]. This study aims to use an evidence-based approach to integrate dimensions from existing IQ frameworks, thus promoting a common understanding of IQ requirements. In addition, safety concerns may discourage health care professionals from adopting DHTs. Although many general practitioners in the United Kingdom would support the deployment of more DHTs in primary care, they are concerned about the safety of digital health information [16]. Thus, there is a need for a framework that can be used to evaluate the extent to which digital health information is suitable for clinical purposes. The aim of this systematic review is to identify and define dimensions within existing IQ frameworks for DHTs and synthesize an IQ framework that can be used to evaluate the extent to which digital health information is fit for clinical purposes, either diagnostic, therapeutic, or prognostic.

## Methods

### Review Checklist

The systematic review is reported based on the PRISMA (Preferred Reporting Items for Systematic Reviews and Meta-Analysis) checklist [17] presented in Multimedia Appendix 1.

### Review Questions

The systematic review will address the following questions:

1. What IQ frameworks currently exist for evaluating DHTs?
2. How are dimensions within these existing IQ frameworks defined?
3. Which IQ dimensions indicate how well digital health information is fit for diagnostic, therapeutic, or prognostic purposes?
4. How are these digital health IQ dimensions related to one another?

### Eligibility Criteria

The eligibility criteria of this review were based on a specific approach for identifying frameworks, theories, and models in a systematic review using behavior of phenomenon of interest, health context and model or theory [18,19]. The traditional population, intervention, comparator, and outcome approach was not suitable as we synthesized frameworks rather than interventions.

We included IQ frameworks for assessing DHTs used for clinical purposes but excluded frameworks for nonclinical or administrative purposes because they are less likely to affect patient safety. For example, an incidence reporting system within a hospital setting can be used for administrative purposes. Similarly, we excluded IQ frameworks for web-based health-related information and electronic learning because they are not directly used in the clinical management of patients at the point of care. We excluded self-management apps used by patients mainly for health education and disease tracking purposes, as their IQ requirements are probably different from those used for clinical purposes by health care professionals [20]. We included multidimensional frameworks, but not individual IQ dimensions, as IQ is an interrelated multidimensional concept. Both published and gray literature were included. The included studies were not restricted based on publication date, and all eligible studies until October 2019 were included. Restrictions based on publication status, study type, and publication date may inadvertently lead to the exclusion of potentially relevant IQ frameworks. A summary of the eligibility criteria is presented in Table 1.

**Table 1:** Inclusion and exclusion criteria.

Concept	Inclusion	Exclusion
Behavior of phenomenon of interest	Information quality or data quality	Information quality or data quality of administrative and nonclinical data
Health context	Use of digital health information for clinical purposes (ie, diagnostic, therapeutic, or prognostic)	Web-based search for health-related information, electronic learning, and digital health apps for self-management
Model or theory	Multidimensional framework	Individual dimension
Language	English	Non-English
Publication status	Published and gray literature	None
Date of publication	Any	None
Type of study	Any	None

### Information Sources

We searched bibliographic health care databases, including Embase, MEDLINE, PubMed, CINAHL, Maternity and Infant Care, PsycINFO, and Global Health. We also searched Scopus to identify digital health publications in non–health care disciplines, such as engineering and computer science. In addition, we searched HMIC (the Health Management Information Consortium) and ProQuest Dissertations and Theses Global, which are regarded as good sources of gray literature [21,22]. We manually searched the references of the included studies and tracked their citations to identify other eligible studies using Scopus and Google Scholar.

### Search Strategy

The search terms are related to 3 main concepts: (1) IQ (behavior of the phenomenon of interest), (2) digital health (health context), and (3) framework (model or theory) [18,19]. The search terms relating to each of these concepts were combined using the *OR* connector. The results of the 3 categories were then combined using the *AND* connector. A librarian was consulted for input on the search strategy. Medical Subject Headings and free-text terms were used. Truncation and adjacency searching were used to increase the sensitivity of the search, as appropriate. The search strategy is presented in Multimedia Appendix 2.

### Data Management

We removed duplicates using Endnote Reference Management Software (Clarivate), and additional duplicates not identified by the Endnote function were removed manually. The deduplicated data were then imported into Covidence (Veritas Health Innovation Ltd), a review-management software program that operates in partnership with Cochrane Collaboration and allows multiple reviewers to work on study selection simultaneously and independently.

### Study Selection

The eligible studies were identified in 2 stages: title and abstract screening and full-text review. Titles and abstracts of the studies were screened for eligibility by 2 independent reviewers (KPF and JTA) using the criteria outlined in Table 1. Conflicts were resolved by discussion between the 2 reviewers and adjudicated by a third independent reviewer (JOD) when necessary. The full-text review of all studies selected during the screening was independently conducted by 2 reviewers (KPF and SOC), with disagreement resolved as described previously.

### Data Extraction

Overall, 2 reviewers (KPF and SOC) independently extracted data from each eligible study using a prepiloted Microsoft Excel data extraction form. Other reviewers (JOD, CC, PAW, JG, JC, and AM) reviewed the extracted data to ensure the accuracy and completeness of the data. We extracted the study details, including authors, year of publication, country, affiliation, study aim, study design, and publication status. We also extracted IQ framework–related data, including the method of framework development, method of framework validation (when available), type of DHT, IQ dimensions and their verbatim definition, categories of IQ dimensions (when available), and metrics of IQ dimension measurement (when available).

These data elements were defined as follows:

- IQ frameworks for DHTs: A systematic integration of IQ dimensions to evaluate health information technologies used in the diagnosis, treatment, and prognosis of patients.
- IQ dimensions within the frameworks in digital health: These are the evaluation criteria within the IQ frameworks that specify the extent to which health information technologies are fit for clinical use.
- Definition of IQ dimensions in digital health: A clear description of what aspect of information each dimension assesses.
- Categories of dimensions within IQ frameworks in digital health: IQ dimensions are often categorized to depict the relationship between IQ dimensions in an IQ framework.
- Metrics of measurement of IQ dimensions in digital health: How each IQ dimension is measured, for example, questionnaire and mathematical formulas.

### Quality Assessment

We assessed the quality of the included studies using the Critical Appraisal Skills Programme (CASP) checklist for qualitative studies [23]. Selecting this tool was difficult, as the included papers comprised a range of methodologies, including ethnography study, literature review, practice brief, and framework development, with some of the papers not explicitly stating their methodology. Therefore, some of the questions on the checklist were not applicable. Scores were not assigned, as this was not recommended by the checklist [23]. Studies were not excluded based on quality assessment outcome, as this was unlikely to have any major impact on the ultimate definition of the dimensions and the resulting IQ framework. However, the assessment provided a general idea about the quality of the development processes of the existing IQ frameworks and, therefore, the strength of the evidence [24].

### Data Synthesis

In this review, the IQ framework was developed using a thematic synthesis approach comprising 3 key stages: coding, descriptive synthesis, and analytical synthesis [25]. Although codes and descriptive themes were generated directly from the extracted definition of IQ dimensions, analytical themes were interpretations that went beyond the original data.

In the first stage, we coded the verbatim definitions of IQ dimensions extracted from the existing IQ frameworks in the included papers. Coding was done by identifying the unique concepts from each definition of the IQ dimension and highlighting them using the text highlight function of Microsoft Word (Microsoft).

Second, we categorized the codes based on their similarities and differences and created a descriptive theme to capture the meaning of each category. Each descriptive theme was defined based on the meaning of the original code from which it was created. The descriptive themes created were regarded as the IQ dimensions of the new IQ framework for digital health. Coding and descriptive synthesis were performed by 2 independent reviewers (KPF and JTA) with adjudication by a third independent reviewer (JOD).

Finally, we conceptualized analytical themes by considering the interrelationship between the descriptive themes (IQ dimensions) based on their definitions. The conceptualization of the analytical themes from the descriptive themes in thematic synthesis has been described as controversial because it is influenced by the insight and judgment of the reviewers [25]. This stage was quite challenging because of the subjective nature and varying perspectives of the reviewers. The following procedures were used to avoid bias and to achieve a consensus. The lead author (KPF) categorized the IQ dimensions without revealing his proposed categories to other reviewers. The other reviewers were then invited to categorize the IQ dimensions individually and email their suggested categories with rationale to the lead author without copying other members of the team. The reviewers were specifically asked to reflect on the suitability of digital health information for clinical purposes and its impact on patient safety while categorizing the IQ dimensions. Overall, 2 reviewers (KPF and JOD) then collated the inputs and carefully assigned a category to each of the dimensions based on the most popular suggestions considered along with the rationale. The framework was then shared with all the members of the team for further inputs and adaptation, if necessary.

Thus, a new digital health IQ framework was developed by synthesizing existing IQ frameworks for DHTs. The IQ dimensions in the new framework are descriptive themes that were generated directly from the definition of IQ dimensions within existing frameworks, whereas the IQ categories were generated from the higher-order analytical synthesis of the descriptive themes.

### Ethics

Ethical approval was not required for this systematic review, as the primary data were not collected. The review was registered in PROSPERO [26], and the protocol was published [27] to promote transparency.

## Results

### Selection of Studies

**Figure 1:**
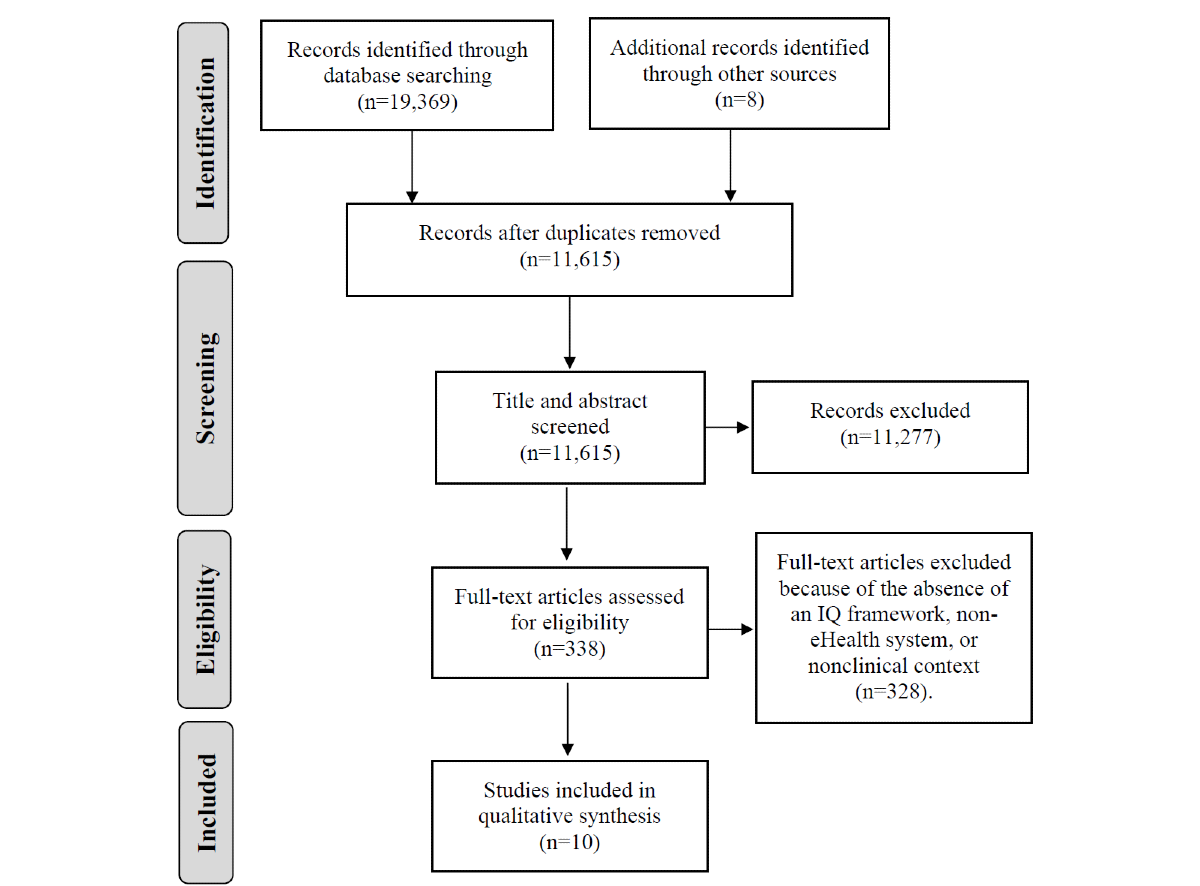
PRISMA (Preferred Reporting Items for Systematic Reviews and Meta-Analysis) flow diagram. IQ: information quality.

A total of 19,377 records were identified from the literature search. These were reduced to 338 after the removal of duplicates and screening of titles and abstracts. Only 10 papers were included in the study after a full-text review. Although 3 of these papers [14,28,29] were in the context of secondary use of digital health data for research, they were included, as their IQ frameworks were relevant to the clinical context of digital health information. However, we performed a sensitivity analysis by conducting a thematic synthesis with and without these 3 papers [30]. The sensitivity analysis revealed that the inclusion of the 3 papers did not affect the component dimensions in the resulting framework, but their inclusion produced a better understanding of the definition of the dimensions. The PRISMA flow diagram is shown in Figure 1.

### Included Papers

The 10 included papers were published between 2007 and 2017. Of the 10 papers, 5 (50%) were published in the United States [14,28,31-33], 3 (30%) were published in the United Kingdom [13,29,34], and one each was published in Canada [35] and Japan [36]. One of the studies published in the United Kingdom was conducted in Saudi Arabia [13]. Of the 10 papers, 4 (40%) were journal publications [14,28,32,36], 3 (30%) were conference papers [29,31,34], 2 (20%) were institutional reports [33,35], and 1 (10%) was a PhD thesis [13]. Of the 10 studies, 5 used qualitative methods, either alone [31,36] or in combination with other methods [13,14,29]. Similarly, 40% (4/10) studies used literature review alone [28,34] or combined with other methods [13,14]. Overall, 30% (3/10) studies modified the existing frameworks [29,32,36]. One study reported to have updated the previous framework [33], but it was unclear how this was achieved. In addition, 10% (1/10) study [35] did not state how the framework was developed. About 50% (5/10) of the frameworks were on EHRs [13,14,28,32,33], one each on electronic medical records [35], primary care databases [29], CDSSs [31], mobile and web-based apps for telemedicine [36], and cloud-based health information systems [34]. Thus, it appears that IQ framework research is unable to keep pace with the rapid evolution of DHTs with obvious underrepresentation of newer DHTs such as mobile health. The details of the included papers are presented in Multimedia Appendix 3 [13,14,28,29,31-36].

### Quality of Included Studies

The quality assessment indicated that most of the studies described an IQ framework for DHT without reporting a robust framework development process. The Critical Appraisal Skills Programme checklist is not applicable to 2 studies [33,35], which are institutional publications. Only 1 qualitative study [13] reported on the recruitment strategy. Similarly, studies with literature reviews did not report on the search strategy or study selection process [13,14,28,34]. Only 3 studies [13,31,32] addressed ethical issues and reported sufficiently rigorous data analysis. These findings further justify the need for this study, which used a robust systematic review approach to develop a preliminary IQ framework for digital health. The quality assessment results are provided in Multimedia Appendix 4 [13,14,28,29,31-36].

### Clinical Information Quality Framework for Digital Health

**Figure 2:**
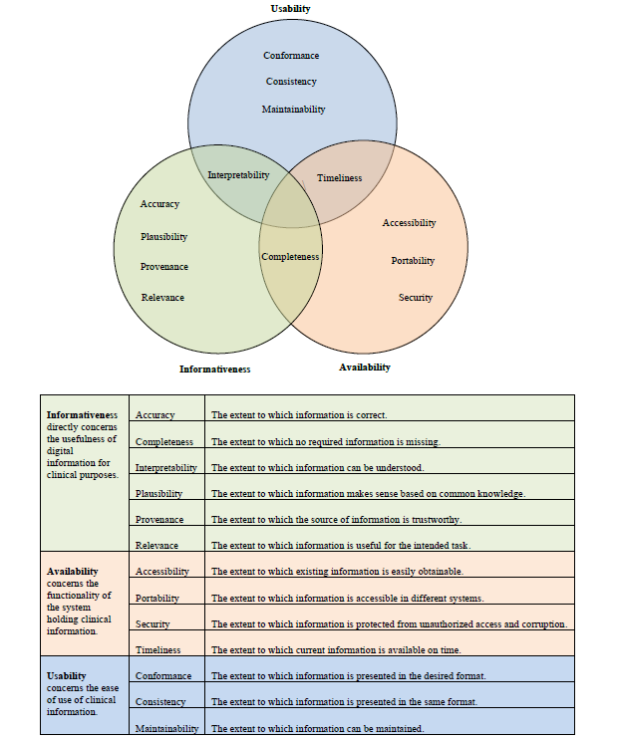
Clinical Information Quality framework for digital health.

A total of 38 IQ dimensions and 70 verbatim definitions were extracted from the 10 included frameworks. The list of dimensions and their definitions are provided in Multimedia Appendix 5 [13,14,28,29,31-36]. The coding of these definitions led to the identification of 160 codes. Aggregation of similar codes resulted in a total of 13 unique IQ dimensions that mirrored all the relevant dimensions in the existing IQ frameworks while eliminating related but redundant dimensions. The resulting dimensions include accessibility, completeness, portability, security, timeliness, accuracy, interpretability, plausibility, provenance, relevance, conformance, consistency, and maintainability. These dimensions were defined based on the codes from which they were generated and classified into higher categories of availability, informativeness, and usability during the analytical synthesis. It is worth noting that some of the dimensions fit into more than one category but were placed into the best-fit category after carefully considering the inputs of all reviewers. For example, completeness was considered fit for both the availability and informativeness categories but was placed in the informativeness category, as this was the most popular category suggested by the reviewers. Similarly, timeliness was considered more fit for the availability category compared with the usability category, and interpretability was placed in the informativeness category rather than the usability category. The resulting Clinical Information Quality (CLIQ) framework for digital health is shown in Figure 2.

### Metrics of Measurement

Metrics of measurement for the IQ dimensions were given in only 30% (3/10) of the included papers [13,29,35]. The remaining papers only conceptualize IQ without providing guidance on its measurement. Objective and subjective measures were used in these studies. Objective measures involved mathematical calculations, such as ratio, percentages, and fraction to quantify the IQ dimension [37]. Subjective measures, on the other hand, rely on the perspectives of the information users, which are usually assessed using a Likert scale questionnaire or qualitative interviews [10].

Objective measures were reported for accuracy, validity, timeliness, completeness and interpretability, comprehensibility, reliability, validity, timeliness, relevance, integrity completeness, concordance, informative sufficiency, consistency, consistency of capture, and consistency of form [13,29,35]. These dimensions were measured by determining whether a desired or undesired attribute was present or absent. For example, Almutiry [13] identified quality problems (undesired attributes) related to accuracy as misspelling, out-of-range values, erroneous values, etc. The quality score for accuracy was then calculated by determining the proportion of the total data units without each quality problem. Similarly, Dungey et al [29] measured accuracy by calculating the proportion of implausible values (undesired attributes). Bowen [35], on the other hand, used the percentage of data units with the desired attribute. Correctness was measured by determining the positive predictive value, which is the proportion of true positives (desired attributes).

Subjective measures were reported for usability, relevance, provenance, secure access, confidentiality, and privacy [13]. Each dimension was measured using multiple Likert scale questions. For example, relevance was assessed by the information users’ rating of how far the information was relevant, useful, applicable, and appropriate to the task at hand [13]. The quality score for each dimension is the aggregate of all ratings for these different measures.

## Discussion

### Principal Findings

We identified 10 existing IQ frameworks for DHTs, and from these, we developed the CLIQ framework for digital health with 13 unique dimensions, including accessibility, completeness, portability, security, timeliness, accuracy, interpretability, plausibility, provenance, relevance, conformance, consistency, and maintainability, which were classified into 3 meaningful categories—availability, informativeness, and usability—based on our conceptualization of *fitness* of digital health information for clinical purposes.

The informativeness category directly concerns the usefulness of information for clinical purposes and has the greatest implications for patient safety. Problems with the dimensions in the category can directly lead to significant harm, as previously reported in the literature [6,8]. Accuracy is the most popular IQ dimension. However, this systematic review echoes the literature that IQ is not only about accuracy but also a multidimensional phenomenon [38]. Provenance and plausibility are unique IQ dimensions that can be regarded as proxies for accuracy, especially in situations where immediate and objective determination of accuracy is impractical. Provenance and plausibility can be easily determined subjectively. For example, knowing that the source of digital health information is a reputable institution (provenance) such as the World Health Organization would be reassuring, and an implausible value, such as a body temperature of 100°C (plausibility), would raise a serious concern. Interpretability is critical to the clinical use of digital information, as an incorrect interpretation may lead to significant harm. Hence, the inclusion of reference values with most laboratory results enhances the safe interpretation of the values.

The availability category of IQ dimensions concerns the functionality of a system that holds clinical information. These dimensions are critical as they can affect the efficiency of service delivery and are regarded as important by users of digital health information. Inaccessible digital information offers no real value to health professionals, as it cannot be used in the clinical management of patients. In addition, accessibility of clinical information wherever it is required (portability) and whenever it is required (timeliness) could be lifesaving, especially in emergency situations when the knowledge of a patient’s medical history and current medications are essential. Timeliness, in the clinical context of digital health, also requires that health information is up to date. On the other hand, restriction of access to clinical information only to authorized users (security) protects the privacy and confidentiality of the patient and protects the information from corruption. Availability dimensions are illustrated by the UK’s Summary Care Records [39], which contain up-to-date personal medical and medication history of patients and are accessible at the point of care (timeliness and accessibility) across different health care settings only to authorized health care professionals (security).

The usability category concerns the ease of use of health information. Consistency and conformance are akin to 2 sides of a coin, with consistency referring to the presentation of information in the same format within a system and conformance referring to the presentation of information in the desired format based on local guidelines or international standards. For example, it is important for an app to present blood glucose consistently using either gram per deciliter or millimoles per liter and conform with the recommended units in the local guidelines to avoid confusion, which may compromise patient safety. The last dimension in this category is maintainability. This refers to the extent to which the information can be maintained. Maintenance, in this context, covers a range of activities, including review, audit, update, and storage of clinical information to ensure that all other IQ requirements are met. For example, timeliness can be improved by updating the information in the DHTs, and accuracy can be improved through regular audits of the information generated by the DHTs.

### Strength and Limitations

The main strength of our framework lies in the rigorous systematic review approach that was used to identify, define, and categorize IQ dimensions. In addition, our approach of synthesizing definitions rather than the traditional practice of simply cross-matching dimensions from different frameworks is more meaningful, as the definition expresses the real meaning of each dimension, and a dimension usually has heterogeneous definitions across different frameworks. In addition, focusing on the clinical context rather than the ever-changing DHTs, as in previous frameworks, we have developed a context-specific IQ framework that would be applicable or at least adaptable to a range of DHTs used in the clinical context, including novel ones that are currently underrepresented in IQ framework research. This approach differs from previous frameworks that focus on individual DHTs, such as EHR [13] and CDSS [31]. The consideration of the clinical purposes of DHTs is in consonance with the *fit-for-purpose* definition of IQ [10]. Moreover, the traditional practice of using the same clinical information across different DHTs (eg, the use of EHR information for CDSS) further justifies the need for a common IQ framework for the clinical context of DHTs.

However, the lack of information about the relative relevance of the IQ dimensions in the CLIQ framework and the optimal means of their measurement are limitations. Although these dimensions could be considered as indices of fitness of digital information for clinical purposes, we acknowledge the need to consult with clinical information users, such as doctors, nurses, and health service managers, as recommended in the literature [10,12]. Thus, the current CLIQ framework for digital health could be regarded as a preliminary framework to be tested in primary research studies. To build on this preliminary research, an international eDelphi study is currently underway to obtain consensus among clinicians on the approach to assessing the quality of clinical information produced by DHTs. The eDelphi study addresses the prioritization of the dimensions and the metrics for measuring the dimension.

### Comparison With Validated IQ Frameworks

The CLIQ framework for digital health shares several characteristics with validated IQ frameworks within and beyond the health care domain. One such validated IQ framework, developed by Wang and Strong [38], has been used as a reference point in IQ research. Out of its 15 dimensions, 7 (accuracy, relevance, completeness, timeliness, interpretability, security, and accessibility) are also included in the CLIQ framework. The rest of its dimensions, such as believability and understandability, were assimilated by other dimensions in our framework during thematic synthesis. On the other hand, novel dimensions such as portability and maintainability are included in our framework but not in the framework developed by Wang and Strong [38]. This reflects technological advances in the last three decades, with an increasing amount of digital information. In addition, our framework was developed for the clinical context, whereas Wang and Strong focused on the business domain [38].

Similarly, the dimensions in our framework overlap with the product quality properties of the International Organization for Standardization/International Electrotechnical Commission (ISO/IEC 25010), which include 8 characteristics: functional suitability, reliability, performance efficiency, operability, security, compatibility, maintainability, and transferability [40]. Of these characteristics, 2 (maintainability and security) were also included in the CLIQ framework. Other dimensions in the CLIQ framework (eg, availability, accuracy, and completeness) are included as subcharacteristics of product quality. This overlap is not unexpected, as DHTs are also software products, with IQ being a subset of product quality [40]. However, ISO/IEC 25010 addresses Systems and Software Quality Requirements and Evaluation from a computer engineering perspective, whereas the CLIQ framework addresses IQ from a health care perspective with consideration of its impact on patient safety [6-8]. Although we recognize the importance of other aspects of product quality, such as user-interface esthetics, these are beyond the scope of this study, which is focused on IQ in the clinical context of DHTs.

### Conclusions

This systematic review highlighted the importance of the IQ of DHTs and their relevance to patient safety. Future research is needed to determine the relative relevance of each dimension in the CLIQ framework and their metrics of measurement, with inputs from clinical information users. The CLIQ framework for digital health will be useful to health care organizations, health care professionals, digital health solution developers, and medical device regulators in conceptualizing and evaluating IQ issues associated with digital health, thus forestalling potential patient safety problems. This is more relevant than ever, as the health care community is increasingly turning to DHTs, and the need for and value of such systems in the context of health emergencies is becoming ever more apparent.

## Appendix

Multimedia Appendix 1 PRISMA (Preferred Reporting Items for Systematic Reviews and Meta-Analysis) checklist.

Multimedia Appendix 2 Search strategy.

Multimedia Appendix 3 Included papers.

Multimedia Appendix 4 Quality assessment result.

Multimedia Appendix 5 Definition of dimensions within the existing information quality frameworks for digital health technologies.

## Abbreviations

CDSS: clinical decision support system
CLIQ: Clinical Information Quality
DHT: digital health technology
EHR: electronic health record
IQ: information quality
PRISMA: Preferred Reporting Items for Systematic Reviews and Meta-Analysis
