# Supplementary material for: Information Quality Frameworks for Digital Health Technologies: Systematic Review
Source: J Med Internet Res. 2021 May 17;23(5):e23479. doi: 10.2196/23479 (PMC8167621; doi:10.2196/23479)
Supplement: Multimedia Appendix 2 [file jmir_v23i5e23479_app2.docx]

**Multimedia Appendix 2: Search Strategy**

Database: **HMIC Health Management Information Consortium** 1979 to July 2019

| **#** | **Searches** | **Results** |
| --- | --- | --- |
| 1 | (information adj1 quality).ab,ti. | 254 |
| 2 | (data adj1 quality).ab,ti. | 435 |
| 3 | (information adj1 accura$).ab,ti. | 180 |
| 4 | (data adj1 accura$).ab,ti. | 126 |
| 5 | (information adj1 reliab$).ab,ti. | 123 |
| 6 | (data adj1 reliab$).ab,ti. | 115 |
| 7 | (information adj1 valid$).ab,ti. | 33 |
| 8 | (data adj1 valid$).ab,ti. | 88 |
| 9 | framework$.ab,ti. | 13858 |
| 10 | model$.ab,ti. | 24453 |
| 11 | scale$.ab,ti. | 7686 |
| 12 | dimension$.ab,ti. | 3031 |
| 13 | indicator$.ab,ti. | 6439 |
| 14 | guideline$.ab,ti. | 9796 |
| 15 | metric$.ab,ti. | 407 |
| 16 | criteri$.ab,ti. | 8085 |
| 17 | standard$.ab,ti. | 21343 |
| 18 | (ehealth or e-health).ab,ti. | 246 |
| 19 | electronic health.ab,ti. | 449 |
| 20 | (telemedicine or tele-medicine).ab,ti. | 828 |
| 21 | (teleconsult$ or tele-consult$).ab,ti. | 126 |
| 22 | (telehealth or tele-health).ab,ti. | 353 |
| 23 | (telemonitor$ or tele-monitor$).ab,ti. | 107 |
| 24 | (telecardiology or tele-cardiology).ab,ti. | 9 |
| 25 | "remote consultation".ab,ti. | 9 |
| 26 | (telecommunication$ or tele-communication$).ab,ti. | 253 |
| 27 | ("video conferenc$" or videoconferenc$ or video-conferenc$).ab,ti. | 225 |
| 28 | telephone$.ab,ti. | 3424 |
| 29 | (teleconferenc$ or tele-conferenc$).ab,ti. | 27 |
| 30 | (mHealth or m-Health or "mobile Health").ab,ti. | 78 |
| 31 | ("mobile telephone$" or "mobile phone$").ab,ti. | 258 |
| 32 | ("cell$ phone$" or cellphone$ or cell-phone$).ab,ti. | 43 |
| 33 | ("smart phone$" or "smartphone$" or smart-phone$).ab,ti. | 85 |
| 34 | ("handheld computer$" or "hand-held computer$").ab,ti. | 43 |
| 35 | ("tablet computer$" or "tablet PC").ab,ti. | 11 |
| 36 | "personal digital assistant$ .".ab,ti. | 32 |
| 37 | "mobile app$".ab,ti. | 23 |
| 38 | "medical app$".ab,ti. | 131 |
| 39 | "health app$".ab,ti. | 325 |
| 40 | "short messag$ service$".ab,ti. | 41 |
| 41 | ("text messag$" or text-messag$).ab,ti. | 115 |
| 42 | ("mp3 player$" or mp3-player$).ab,ti. | 1 |
| 43 | ("handheld device$" or "hand-held device$").ab,ti. | 13 |
| 44 | "mobile device$".ab,ti. | 28 |
| 45 | "software app$".ab,ti. | 22 |
| 46 | "computer-assisted decision$".ab,ti. | 6 |
| 47 | (CDSS or CDS).ab,ti. | 120 |
| 48 | "decision support system$".ab,ti. | 230 |
| 49 | "computer app$".ab,ti. | 92 |
| 50 | "computer software$".ab,ti. | 118 |
| 51 | "computer-assisted instruction$".ab,ti. | 13 |
| 52 | "clinical decision support".ab,ti. | 92 |
| 53 | "health information system$".ab,ti. | 192 |
| 54 | "medical information system$".ab,ti. | 55 |
| 55 | "laboratory information system$".ab,ti. | 6 |
| 56 | "electronic health record$".ab,ti. | 391 |
| 57 | "electronic medical record$".ab,ti. | 279 |
| 58 | "clinical information system$".ab,ti. | 181 |
| 59 | "computerized medical record$".ab,ti. | 19 |
| 60 | EHR.ab,ti. | 162 |
| 61 | "hospital information system$".ab,ti. | 176 |
| 62 | internet.ab,ti. | 1918 |
| 63 | "social media".ab,ti. | 164 |
| 64 | blog$.ab,ti. | 47 |
| 65 | facebook.ab,ti. | 45 |
| 66 | (twitter or tweet$).ab,ti. | 53 |
| 67 | instagram.ab,ti. | 0 |
| 68 | e-mail.ab,ti. | 224 |
| 69 | ("electronic prescri$" or eprescri$ or e-prescri$).ab,ti. | 189 |
| 70 | computer$ physician order entry.ab,ti. | 29 |
| 71 | "health 2.0".ab,ti. | 9 |
| 72 | (web or website$).ab,ti. | 5405 |
| 73 | (email or "electronic mail").ab,ti. | 356 |
| 74 | linkedin.ab,ti. | 4 |
| 75 | (computeri#ed medical record$ or computeri#ed health record$).ab,ti. | 60 |
| 76 | (EHR or EMR or "electronic patient record$").ab,ti. | 649 |
| 77 | (fitbit or wearable$).ab,ti. | 23 |
| 78 | ("patient health record$" or PHR).ab,ti. | 26 |
| 79 | "information audit$".ab,ti. | 16 |
| 80 | "data audit$".ab,ti. | 11 |
| 81 | measur$.ab,ti. | 32286 |
| 82 | Computer software/ | 521 |
| 83 | Computer applications/ | 1101 |
| 84 | Decision support systems/ | 216 |
| 85 | Information systems/ | 2448 |
| 86 | Health records/ or electronic patient records/ | 1580 |
| 87 | Medical information systems/ | 380 |
| 88 | Computerised information systems/ | 132 |
| 89 | District information support systems/ | 4 |
| 90 | Drug information systems/ | 122 |
| 91 | Health service information systems/ | 34 |
| 92 | Medical information systems/ | 380 |
| 93 | Online information systems/ | 197 |
| 94 | telemedicine/ | 1307 |
| 95 | telecare/ | 723 |
| 96 | Mobile telephones/ | 279 |
| 97 | Telephones/ | 45 |
| 98 | Telecommunications/ | 241 |
| 99 | Videoconferencing/ | 28 |
| 100 | Personal digital assistants/ | 2 |
| 101 | Text messaging/ | 84 |
| 102 | Email/ | 147 |
| 103 | Electronic prescribing/ | 185 |
| 104 | Internet portals/ or Internet/ or Internet websites/ | 2153 |
| 105 | Broadband/ | 2 |
| 106 | Electronic data interchange/ | 56 |
| 107 | World Wide Web/ | 103 |
| 108 | Lifelong electronic health records/ | 54 |
| 109 | Frameworks/ | 1354 |
| 110 | 1 or 2 or 3 or 4 or 5 or 6 or 7 or 8 or 79 or 80 | 1329 |
| 111 | 9 or 10 or 11 or 12 or 13 or 14 or 15 or 16 or 17 or 109 | 77585 |
| 112 | 18 or 19 or 20 or 21 or 22 or 23 or 24 or 25 or 26 or 27 or 28 or 29 or 30 or 31 or 32 or 33 or 34 or 35 or 36 or 37 or 38 or 39 or 40 or 41 or 42 or 43 or 44 or 45 or 46 or 47 or 48 or 49 or 50 or 51 or 52 or 53 or 54 or 55 or 56 or 57 or 58 or 59 or 60 or 61 or 62 or 63 or 64 or 65 or 66 or 67 or 68 or 69 or 70 or 71 or 72 or 73 or 74 or 75 or 76 or 77 or 78 or 79 or 80 or 81 or 82 or 83 or 84 or 85 or 86 or 87 or 88 or 89 or 90 or 91 or 92 or 93 or 94 or 95 or 96 or 97 or 98 or 99 or 100 or 101 or 102 or 103 or 104 or 105 or 106 or 107 or 108 or 109 | 51539 |
| 113 | skype.mp. | 10 |
| 114 | 112 or 113 | 51540 |
| 115 | 110 and 111 and 114 | 270 |

Database: **Maternity & Infant Care Database (MIDIRS)**1971 to September 2019

| **#** | **Searches** | **Results** |
| --- | --- | --- |
| 1 | (information adj1 quality).ab,ti. | 68 |
| 2 | (data adj1 quality).ab,ti. | 267 |
| 3 | (information adj1 accura$).ab,ti. | 254 |
| 4 | (data adj1 accura$).ab,ti. | 103 |
| 5 | (information adj1 reliab$).ab,ti. | 113 |
| 6 | (data adj1 reliab$).ab,ti. | 85 |
| 7 | (information adj1 valid$).ab,ti. | 21 |
| 8 | (data adj1 valid$).ab,ti. | 90 |
| 9 | framework$.ab,ti. | 3745 |
| 10 | model$.ab,ti. | 20986 |
| 11 | scale$.ab,ti. | 9587 |
| 12 | dimension$.ab,ti. | 3064 |
| 13 | indicator$.ab,ti. | 4272 |
| 14 | guideline$.ab,ti. | 10676 |
| 15 | metric$.ab,ti. | 330 |
| 16 | criteri$.ab,ti. | 11775 |
| 17 | standard$.ab,ti. | 17276 |
| 18 | (ehealth or e-health).ab,ti. | 38 |
| 19 | electronic health.ab,ti. | 187 |
| 20 | (telemedicine or tele-medicine).ab,ti. | 89 |
| 21 | (teleconsult$ or tele-consult$).ab,ti. | 6 |
| 22 | (telehealth or tele-health).ab,ti. | 22 |
| 23 | (telemonitor$ or tele-monitor$).ab,ti. | 8 |
| 24 | (telecardiology or tele-cardiology).ab,ti. | 3 |
| 25 | "remote consultation".ab,ti. | 4 |
| 26 | (telecommunication$ or tele-communication$).ab,ti. | 24 |
| 27 | ("video conferenc$" or videoconferenc$ or video-conferenc$).ab,ti. | 34 |
| 28 | telephone$.ab,ti. | 2060 |
| 29 | (teleconferenc$ or tele-conferenc$).ab,ti. | 23 |
| 30 | (mHealth or m-Health or "mobile Health").ab,ti. | 80 |
| 31 | ("mobile telephone$" or "mobile phone$").ab,ti. | 136 |
| 32 | ("cell$ phone$" or cellphone$ or cell-phone$).ab,ti. | 56 |
| 33 | ("smart phone$" or "smartphone$" or smart-phone$).ab,ti. | 114 |
| 34 | ("handheld computer$" or "hand-held computer$").ab,ti. | 4 |
| 35 | ("tablet computer$" or "tablet PC").ab,ti. | 4 |
| 36 | "personal digital assistant$ .".ab,ti. | 14 |
| 37 | "mobile app$".ab,ti. | 48 |
| 38 | "medical app$".ab,ti. | 55 |
| 39 | "health app$".ab,ti. | 117 |
| 40 | "short messag$ service$".ab,ti. | 23 |
| 41 | ("text messag$" or text-messag$).ab,ti. | 119 |
| 42 | ("mp3 player$" or mp3-player$).ab,ti. | 3 |
| 43 | ("handheld device$" or "hand-held device$").ab,ti. | 5 |
| 44 | "mobile device$".ab,ti. | 23 |
| 45 | "software app$".ab,ti. | 8 |
| 46 | "computer-assisted decision$".ab,ti. | 3 |
| 47 | (CDSS or CDS).ab,ti. | 89 |
| 48 | "decision support system$".ab,ti. | 34 |
| 49 | "computer app$".ab,ti. | 6 |
| 50 | "computer software$".ab,ti. | 47 |
| 51 | "computer-assisted instruction$".ab,ti. | 8 |
| 52 | "clinical decision support".ab,ti. | 29 |
| 53 | "health information system$".ab,ti. | 125 |
| 54 | "medical information system$".ab,ti. | 6 |
| 55 | "laboratory information system$".ab,ti. | 4 |
| 56 | "electronic health record$".ab,ti. | 163 |
| 57 | "electronic medical record$".ab,ti. | 305 |
| 58 | "clinical information system$".ab,ti. | 13 |
| 59 | "computerized medical record$".ab,ti. | 11 |
| 60 | EHR.ab,ti. | 46 |
| 61 | "hospital information system$".ab,ti. | 18 |
| 62 | internet.ab,ti. | 1156 |
| 63 | "social media".ab,ti. | 254 |
| 64 | blog$.ab,ti. | 97 |
| 65 | facebook.ab,ti. | 124 |
| 66 | (twitter or tweet$).ab,ti. | 58 |
| 67 | instagram.ab,ti. | 4 |
| 68 | e-mail.ab,ti. | 207 |
| 69 | ("electronic prescri$" or eprescri$ or e-prescri$).ab,ti. | 8 |
| 70 | computer$ physician order entry.ab,ti. | 24 |
| 71 | "health 2.0".ab,ti. | 1 |
| 72 | Decision support systems.de. | 29 |
| 73 | Telemedicine.de. | 46 |
| 74 | Telephone.de. | 37 |
| 75 | Mobile phone.de. | 1 |
| 76 | Computers - handheld.de. | 7 |
| 77 | Mobile applications.de. | 25 |
| 78 | Software.de. | 34 |
| 79 | Mobile telephone.de. | 57 |
| 80 | Text messaging.de. | 20 |
| 81 | Internet.de. | 891 |
| 82 | Social media.de. | 142 |
| 83 | Internet.de. | 891 |
| 84 | Information systems.de. | 134 |
| 85 | Medical records.de. | 602 |
| 86 | Electronic health records.de. | 80 |
| 87 | (web or website$).ab,ti. | 2613 |
| 88 | (email or "electronic mail").ab,ti. | 220 |
| 89 | linkedin.ab,ti. | 4 |
| 90 | (computeri#ed medical record$ or computeri#ed health record$).ab,ti. | 16 |
| 91 | (EHR or EMR or "electronic patient record$").ab,ti. | 142 |
| 92 | (fitbit or wearable$).ab,ti. | 14 |
| 93 | ("patient health record$" or PHR).ab,ti. | 9 |
| 94 | "information audit$".ab,ti. | 3 |
| 95 | "data audit$".ab,ti. | 5 |
| 96 | 1 or 2 or 3 or 4 or 5 or 6 or 7 or 8 or 94 or 95 | 983 |
| 97 | measur$.ab,ti. | 44125 |
| 98 | 9 or 10 or 11 or 12 or 13 or 14 or 15 or 16 or 17 or 97 | 93072 |
| 99 | 18 or 19 or 20 or 21 or 22 or 23 or 24 or 25 or 26 or 27 or 28 or 29 or 30 or 31 or 32 or 33 or 34 or 35 or 36 or 37 or 38 or 39 or 40 or 41 or 42 or 43 or 44 or 45 or 46 or 47 or 48 or 49 or 50 or 51 or 52 or 53 or 54 or 55 or 56 or 57 or 58 or 59 or 60 or 61 or 62 or 63 or 64 or 65 or 66 or 67 or 68 or 69 or 70 or 71 or 72 or 73 or 74 or 75 or 76 or 77 or 78 or 79 or 80 or 81 or 82 or 83 or 84 or 85 or 86 or 87 or 88 or 89 or 90 or 91 or 92 or 93 | 8465 |
| 100 | 96 and 98 and 99 | 70 |

Database: **Embase Classic+Embase**1947 to 2019 October 23

| **#** | **Searches** | **Results** |
| --- | --- | --- |
| 1 | (information adj1 quality).ti,ab,kw. | 2744 |
| 2 | (data adj1 quality).ti,ab,kw. | 16166 |
| 3 | (information adj1 reliab*).ti,ab,kw. | 5495 |
| 4 | (data adj1 reliab*).ti,ab,kw. | 7178 |
| 5 | (information adj1 valid*).ti,ab,kw. | 1382 |
| 6 | (data adj1 valid*).ti,ab,kw. | 9702 |
| 7 | (information adj1 audit*).ti,ab,kw. | 2048 |
| 8 | (data adj1 audit*).ti,ab,kw. | 2127 |
| 9 | (information adj1 accura*).ti,ab,kw. | 7124 |
| 10 | (data adj1 accura*).ti,ab,kw. | 5483 |
| 11 | framework*.ti,ab,kw. | 279717 |
| 12 | dimension*.ti,ab,kw. | 584869 |
| 13 | scale*.ti,ab,kw. | 1042015 |
| 14 | indicator*.ti,ab,kw. | 355689 |
| 15 | guideline*.ti,ab,kw. | 525019 |
| 16 | metric*.ti,ab,kw. | 78947 |
| 17 | measur*.ti,ab,kw. | 4452237 |
| 18 | criteri*.ti,ab,kw. | 1001109 |
| 19 | model*.ti,ab,kw. | 3514436 |
| 20 | standard*.ti,ab,kw. | 1782018 |
| 21 | ehealth.ti,ab,kw. | 3428 |
| 22 | e-health.ti,ab,kw. | 3518 |
| 23 | telemedicine.ti,ab,kw. | 15699 |
| 24 | tele-medicine*.ti,ab,kw. | 243 |
| 25 | teleradio*.ti,ab,kw. | 2123 |
| 26 | tele-radio*.ti,ab,kw. | 80 |
| 27 | telepathology.ti,ab,kw. | 878 |
| 28 | tele-pathology.ti,ab,kw. | 33 |
| 29 | telerehabilitation.ti,ab,kw. | 867 |
| 30 | tele-rehabilitation.ti,ab,kw. | 196 |
| 31 | teleconsult*.ti,ab,kw. | 1470 |
| 32 | tele-consult*.ti,ab,kw. | 169 |
| 33 | (telehealth or tele-health).ti,ab,kw. | 5554 |
| 34 | (telecare or tele-care).ti,ab,kw. | 903 |
| 35 | teledermatology.ti,ab,kw. | 1069 |
| 36 | tele-dermatology.ti,ab,kw. | 29 |
| 37 | telecardiology.ti,ab,kw. | 298 |
| 38 | tele-cardiology.ti,ab,kw. | 31 |
| 39 | "remote consultation*".ti,ab,kw. | 462 |
| 40 | (telemonitor* or tele-monitor*).ti,ab,kw. | 2503 |
| 41 | (telepsychiatry or tele-psychiatry).ti,ab,kw. | 711 |
| 42 | telediagn*.ti,ab,kw. | 251 |
| 43 | tele-diagn*.ti,ab,kw. | 47 |
| 44 | telesurgery.ti,ab,kw. | 350 |
| 45 | tele-surgery.ti,ab,kw. | 57 |
| 46 | teletherapy.ti,ab,kw. | 1949 |
| 47 | tele-therapy.ti,ab,kw. | 29 |
| 48 | Telenursing/ | 258 |
| 49 | Telenursing.ti,ab,kw. | 203 |
| 50 | Tele-nursing.ti,ab,kw. | 31 |
| 51 | Telepharmacy.ti,ab,kw. | 146 |
| 52 | Tele-pharmacy.ti,ab,kw. | 14 |
| 53 | "electronic health".ti,ab,kw. | 22032 |
| 54 | "mobile health".ti,ab,kw. | 3675 |
| 55 | mHealth.ti,ab,kw. | 3378 |
| 56 | m-Health.ti,ab,kw. | 646 |
| 57 | "digital health".ti,ab,kw. | 1460 |
| 58 | ("hand-held computer*" or "handheld computer*").ti,ab,kw. | 845 |
| 59 | ("handheld device*" or "hand-held device*").ti,ab,kw. | 1659 |
| 60 | "tablet computer*".ti,ab,kw. | 1067 |
| 61 | "Tablet PC*".ti,ab,kw. | 391 |
| 62 | "personal digital assistant*".ti,ab,kw. | 1194 |
| 63 | telecommunication*.ti,ab,kw. | 4067 |
| 64 | tele-communication*.ti,ab,kw. | 22 |
| 65 | videoconferenc*.ti,ab,kw. | 2843 |
| 66 | video-conferenc*.ti,ab,kw. | 1195 |
| 67 | "video conference*".ti,ab,kw. | 387 |
| 68 | "cell phone*".ti,ab,kw. | 3443 |
| 69 | "cellular phone*".ti,ab,kw. | 1226 |
| 70 | cellphone*.ti,ab,kw. | 490 |
| 71 | cell-phone*.ti,ab,kw. | 3443 |
| 72 | smartphone*.ti,ab,kw. | 12617 |
| 73 | smart-phone*.ti,ab,kw. | 2208 |
| 74 | "smart phone*".ti,ab,kw. | 2208 |
| 75 | "Short messag* service*".ti,ab,kw. | 1221 |
| 76 | ("Multimedia messag* service*" or "Multi-media messag* service*").ti,ab,kw. | 52 |
| 77 | "MMS".ti,ab,kw. | 6402 |
| 78 | "SMS".ti,ab,kw. | 7545 |
| 79 | "mobile phone*".ti,ab,kw. | 9616 |
| 80 | "Mobile app*".ti,ab,kw. | 4834 |
| 81 | "medical app*".ti,ab,kw. | 13412 |
| 82 | "health app*".ti,ab,kw. | 6873 |
| 83 | ("text messag*" or text-messag*).ti,ab,kw. | 4889 |
| 84 | Mobile Applications/ | 8003 |
| 85 | MP3-Player/ | 192 |
| 86 | MP3 player*.ti,ab,kw. | 185 |
| 87 | MP3-player*.ti,ab,kw. | 185 |
| 88 | internet/ or blogging/ or social media/ | 119607 |
| 89 | internet.ti,ab,kw. | 67816 |
| 90 | blog*.ti,ab,kw. | 2705 |
| 91 | social media.ti,ab,kw. | 13535 |
| 92 | facebook.ti,ab,kw. | 5043 |
| 93 | twitter.ti,ab,kw. | 3847 |
| 94 | Whatsapp.ti,ab,kw. | 526 |
| 95 | Instagram.ti,ab,kw. | 571 |
| 96 | Linked-In.ti,ab,kw. | 5049 |
| 97 | "Medical information system*".ti,ab,kw. | 1201 |
| 98 | "laboratory information system*".ti,ab,kw. | 2284 |
| 99 | "pharmacy information system*".ti,ab,kw. | 177 |
| 100 | "Hospital Information system*".ti,ab,kw. | 3828 |
| 101 | "nursing information system*".ti,ab,kw. | 267 |
| 102 | ("clinical decision Support" or "decision support system").ti,ab,kw. | 9014 |
| 103 | electronic health records/ or medical records systems, computerized/ | 13833 |
| 104 | ("Electronic Health Record*" or "Computeri#ed Health Record*" or EHR).ti,ab,kw. | 22995 |
| 105 | ("Electronic Medical Record*" or "Computeri#ed Medical Record*" or EMR).ti,ab,kw. | 42627 |
| 106 | conceptual framework/ | 24894 |
| 107 | telehealth/ or telemedicine/ or telecardiology/ or teleconsultation/ or teledermatology/ or telediagnosis/ or telemonitoring/ or telepathology/ or telepsychiatry/ or teleradiology/ or teleradiotherapy/ or telesurgery/ or teletherapy/ | 41087 |
| 108 | personal digital assistant/ | 1390 |
| 109 | mobile application/ or mobile phone/ or smartphone/ | 31168 |
| 110 | telecommunication/ or teleconference/ or telehealth/ | 31266 |
| 111 | blogging/ or e-mail/ or internet/ or mobile phone/ or modem/ or social media/ or telecommunication/ or telephone/ or text messaging/ or videoconferencing/ or wireless communication/ | 213341 |
| 112 | decision support system/ or clinical decision support system/ | 23748 |
| 113 | medical information system/ or nursing information system/ | 20468 |
| 114 | hospital information system/ or laboratory information system/ or operating room information system/ or physician order entry system/ or "point of care system"/ or radiology information system/ | 23295 |
| 115 | computerized provider order entry/ or electronic prescribing/ | 4663 |
| 116 | "electronic prescri*".ti,ab,kw. | 2210 |
| 117 | computer assisted radiotherapy/ or computer assisted therapy/ or computer assisted drug therapy/ | 15079 |
| 118 | computer assisted diagnosis/ | 39534 |
| 119 | computer assisted surgery/ | 9197 |
| 120 | computer assisted surgery system/ | 119 |
| 121 | mobile application/ | 9242 |
| 122 | "portable software".ti,ab,kw. | 28 |
| 123 | mp3 player/ | 192 |
| 124 | ("palm-top computer*" or "palmtop computer*").ti,ab,kw. | 142 |
| 125 | skype.ti,ab,kw. | 673 |
| 126 | "Health* information system* ".ab,kw,ti. | 4741 |
| 127 | "clinical information system* ".ab,kw,ti. | 2075 |
| 128 | (PDA or PDAs).ab,kw,ti. | 16202 |
| 129 | ("electronic patient record* " or "patient health record*" or "patient portal" or PHR).ab,kw,ti. | 7242 |
| 130 | ("computer-assisted decision" or "computer-assisted therapy" or "computer-assisted diagnosis" or "computer-assisted surgery").ab,kw,ti. | 3021 |
| 131 | ("electronic order entry" or "computer* physician order entry").ab,kw,ti. | 1514 |
| 132 | (web or website*).ab,kw,ti. | 161579 |
| 133 | ("electronic mail" or email or e-mail).ab,kw,ti. | 26448 |
| 134 | 1 or 2 or 3 or 4 or 5 or 6 or 7 or 8 or 9 or 10 | 58079 |
| 135 | 11 or 12 or 13 or 14 or 15 or 16 or 17 or 18 or 19 or 20 or 106 | 10289384 |
| 136 | 21 or 22 or 23 or 24 or 25 or 26 or 27 or 28 or 29 or 30 or 31 or 32 or 33 or 34 or 35 or 36 or 37 or 38 or 39 or 40 or 41 or 42 or 43 or 44 or 45 or 46 or 47 or 48 or 49 or 50 or 51 or 52 or 53 or 54 or 55 or 56 or 57 or 58 or 59 or 60 or 61 or 62 or 63 or 64 or 65 or 66 or 67 or 68 or 69 or 70 or 71 or 72 or 73 or 74 or 75 or 76 or 77 or 78 or 79 or 80 or 81 or 82 or 83 or 84 or 85 or 86 or 87 or 88 or 89 or 90 or 91 or 92 or 93 or 94 or 95 or 96 or 97 or 98 or 99 or 100 or 101 or 102 or 103 or 104 or 105 or 107 or 108 or 109 or 110 or 111 or 112 or 113 or 114 or 115 or 116 or 117 or 118 or 119 or 120 or 121 or 122 or 123 or 124 or 125 or 126 or 127 or 128 or 129 or 130 or 131 or 132 or 133 | 659761 |
| 137 | 134 and 135 and 136 | 4560 |

Database: **Global Health**1973 to 2019 Week 42

| **#** | **Searches** | **Results** |
| --- | --- | --- |
| 1 | (information adj1 quality).ab,id,ti. | 392 |
| 2 | (data adj1 quality).ab,id,ti. | 2615 |
| 3 | (information adj1 accura$).ab,id,ti. | 933 |
| 4 | (data adj1 accura$).ab,id,ti. | 677 |
| 5 | (information adj1 reliab$).ab,id,ti. | 696 |
| 6 | (data adj1 reliab$).ab,id,ti. | 1050 |
| 7 | (information adj1 audit$).ab,id,ti. | 20 |
| 8 | (data adj1 audit$).ab,id,ti. | 159 |
| 9 | (information adj1 valid$).ab,id,ti. | 182 |
| 10 | (data adj1 valid$).ab,id,ti. | 1192 |
| 11 | 1 or 2 or 3 or 4 or 5 or 6 or 7 or 8 or 9 or 10 | 7736 |
| 12 | framework$.ab,id,ti. | 33867 |
| 13 | model$.ab,id,ti. | 368714 |
| 14 | scale$.ab,id,ti. | 91684 |
| 15 | dimension$.ab,id,ti. | 28457 |
| 16 | indicator$.ab,id,ti. | 64998 |
| 17 | guideline$.ab,id,ti. | 60796 |
| 18 | metric$.ab,id,ti. | 4817 |
| 19 | measur$.ab,id,ti. | 468933 |
| 20 | criteri$.ab,id,ti. | 82616 |
| 21 | standard$.ab,id,ti. | 230183 |
| 22 | (ehealth or e-health).ab,id,ti. | 611 |
| 23 | "electronic health".ab,id,ti. | 1458 |
| 24 | (telemedicine or tele-medicine).ab,id,ti. | 732 |
| 25 | (telenursing or tele-nursing).ab,id,ti. | 11 |
| 26 | (telepharmacy or tele-pharmacy).ab,id,ti. | 13 |
| 27 | (teleconsult$ or tele-consult$).ab,id,ti. | 100 |
| 28 | (telerehabilitation or tele-rehabilitation).ab,id,ti. | 7 |
| 29 | (telehealth or tele-health or telecare or tele-care).ab,id,ti. | 415 |
| 30 | (telediagn$ or tele-diagn$).ab,id,ti. | 17 |
| 31 | (telemonitor$ or tele-monitor$).ab,id,ti. | 59 |
| 32 | (teleradio$ or tele-radio$).ab,id,ti. | 28 |
| 33 | (telepathology or tele-pathology).ab,id,ti. | 21 |
| 34 | (telepsychiatry or tele-psychiatry).ab,id,ti. | 33 |
| 35 | (telecardiology or tele-cardiology).ab,id,ti. | 12 |
| 36 | (teledermatology or tele-dermatology).ab,id,ti. | 57 |
| 37 | (teletherapy or tele-therapy).ab,id,ti. | 18 |
| 38 | "remote consultation".ab,id,ti. | 26 |
| 39 | (telecommunication$ or tele-communication$).ab,id,ti. | 326 |
| 40 | ("video conferenc$" or videoconferenc$ or video-conferenc$).ab,id,ti. | 246 |
| 41 | telephone$.ab,id,ti. | 11223 |
| 42 | (teleconferenc$ or tele-conferenc$).ab,id,ti. | 127 |
| 43 | (mHealth or m-Health or "mobile Health").ab,id,ti. | 825 |
| 44 | ("mobile telephone$" or "mobile phone$").ab,id,ti. | 1996 |
| 45 | ("cell$ phone$" or cellphone$ or cell-phone$).ab,id,ti. | 756 |
| 46 | ("smart phone$" or "smartphone$" or smart-phone$).ab,id,ti. | 1138 |
| 47 | ("handheld computer$" or "hand-held computer$").ab,id,ti. | 110 |
| 48 | ("palmtop computer$" or "palm-top computer$").ab,id,ti. | 11 |
| 49 | ("tablet computer$" or "tablet PC").ab,id,ti. | 86 |
| 50 | ("personal digital assistant$" or PDA or PDAs).ab,id,ti. | 1919 |
| 51 | "mobile app$".ab,id,ti. | 450 |
| 52 | "medical app$".ab,id,ti. | 1038 |
| 53 | "health app$".ab,id,ti. | 2247 |
| 54 | "short messag$ service$".ab,id,ti. | 302 |
| 55 | ("text messag$" or text-messag$).ab,id,ti. | 1165 |
| 56 | ("multimedia messag$ servic$" or "multi-media messag$ servic$").ab,id,ti. | 4 |
| 57 | ("mp3 player$" or mp3-player$).ab,id,ti. | 13 |
| 58 | ("handheld device$" or "hand-held device$").ab,id,ti. | 119 |
| 59 | "mobile device$".ab,id,ti. | 245 |
| 60 | "software app$".ab,id,ti. | 180 |
| 61 | "clinical decision support".ab,id,ti. | 242 |
| 62 | "computer-assisted decision$".ab,id,ti. | 7 |
| 63 | "computer-assisted diagnosis".ab,id,ti. | 4 |
| 64 | "computer-assisted therapy".ab,id,ti. | 1 |
| 65 | (CDSS or CDS).ab,id,ti. | 1027 |
| 66 | "computer-assisted surgery".ab,id,ti. | 10 |
| 67 | "decision support system$".ab,id,ti. | 594 |
| 68 | "computer app$".ab,id,ti. | 898 |
| 69 | "computer software$".ab,id,ti. | 466 |
| 70 | "computer-assisted instruction$".ab,id,ti. | 24 |
| 71 | decision support systems/ or computer software/ | 4706 |
| 72 | "health information system$".ab,id,ti. | 1038 |
| 73 | "medical information system$".ab,id,ti. | 48 |
| 74 | "laboratory information system$".ab,id,ti. | 169 |
| 75 | "pharmacy information system$".ab,id,ti. | 4 |
| 76 | ("electronic health record$" or "electronic patient record$" or EHR or "patient health record$" or PHR).ab,id,ti. | 1674 |
| 77 | ("electronic medical record$" or EMR).ab,id,ti. | 2280 |
| 78 | "clinical information system$".ab,id,ti. | 58 |
| 79 | ("computeri#ed medical record$" or "computeri#ed health record$").ab,id,ti. | 108 |
| 80 | ("wearable$" or fitbit or "digital health").ab,id,ti. | 416 |
| 81 | ("hospital information system$" or "healthcare information system$").ab,id,ti. | 444 |
| 82 | information systems/ | 4182 |
| 83 | medical records/ | 2658 |
| 84 | internet.ab,id,ti. | 8632 |
| 85 | "social network$".ab,id,ti. | 3601 |
| 86 | "social media".ab,id,ti. | 1540 |
| 87 | blog$.ab,id,ti. | 240 |
| 88 | facebook.ab,id,ti. | 559 |
| 89 | (twitter or tweet$).ab,id,ti. | 426 |
| 90 | whatsapp.ab,id,ti. | 48 |
| 91 | instagram.ab,id,ti. | 58 |
| 92 | linked-in.ab,id,ti. | 404 |
| 93 | (e-mail or email or electronic mail).ab,id,ti. | 1711 |
| 94 | ("electronic prescri$" or eprescri$ or e-prescri$).ab,id,ti. | 94 |
| 95 | ("computer$ physician order entry" or "electronic order entry").ab,id,ti. | 40 |
| 96 | (web or website$).ab,id,ti. | 19259 |
| 97 | "medicine 2.0".ab,id,ti. | 17 |
| 98 | "health 2.0".ab,id,ti. | 39 |
| 99 | mobile telephones/ or telephones/ | 3120 |
| 100 | telemedicine/ or telecommunications/ | 1944 |
| 101 | skype.ab,id,ti. | 47 |
| 102 | internet/ or telecommunications/ or web sites/ | 9369 |
| 103 | social media/ | 1628 |
| 104 | 12 or 13 or 14 or 15 or 16 or 17 or 18 or 19 or 20 or 21 | 1106982 |
| 105 | 22 or 23 or 24 or 25 or 26 or 27 or 28 or 29 or 30 or 31 or 32 or 33 or 34 or 35 or 36 or 37 or 38 or 39 or 40 or 41 or 42 or 43 or 44 or 45 or 46 or 47 or 48 or 49 or 50 or 51 or 52 or 53 or 54 or 55 or 56 or 57 or 58 or 59 or 60 or 61 or 62 or 63 or 64 or 65 or 66 or 67 or 68 or 69 or 70 or 71 or 72 or 73 or 74 or 75 or 76 or 77 or 78 or 79 or 80 or 81 or 82 or 83 or 84 or 85 or 86 or 87 or 88 or 89 or 90 or 91 or 92 or 93 or 94 or 95 or 96 or 97 or 98 or 99 or 100 or 101 or 102 or 103 | 70398 |
| 106 | 11 and 104 and 105 | 604 |

Database: **PsycINFO**1806 to October Week 2 2019

| **#** | **Searches** | **Results** |
| --- | --- | --- |
| 1 | (information adj1 quality).ab,id,ti. | 1035 |
| 2 | (data adj1 quality).ab,id,ti. | 1935 |
| 3 | (information adj1 accura$).ab,id,ti. | 1862 |
| 4 | (data adj1 accura$).ab,id,ti. | 767 |
| 5 | (information adj1 reliab$).ab,id,ti. | 1051 |
| 6 | (data adj1 reliab$).ab,id,ti. | 1527 |
| 7 | (information adj1 audit$).ab,id,ti. | 1464 |
| 8 | (data adj1 audit$).ab,id,ti. | 193 |
| 9 | (information adj1 valid$).ab,id,ti. | 654 |
| 10 | (data adj1 valid$).ab,id,ti. | 2462 |
| 11 | 1 or 2 or 3 or 4 or 5 or 6 or 7 or 8 or 9 or 10 | 12711 |
| 12 | framework$.ab,id,ti. | 184547 |
| 13 | model$.ab,id,ti. | 709343 |
| 14 | scale$.ab,id,ti. | 361958 |
| 15 | dimension$.ab,id,ti. | 164666 |
| 16 | indicator$.ab,id,ti. | 64906 |
| 17 | guideline$.ab,id,ti. | 59884 |
| 18 | metric$.ab,id,ti. | 13788 |
| 19 | measur$.ab,id,ti. | 771160 |
| 20 | criteri$.ab,id,ti. | 163125 |
| 21 | standard$.ab,id,ti. | 205140 |
| 22 | models/ | 65035 |
| 23 | (ehealth or e-health).ab,id,ti. | 1636 |
| 24 | "electronic health".ab,id,ti. | 1856 |
| 25 | (telemedicine or tele-medicine).ab,id,ti. | 1880 |
| 26 | (telenursing or tele-nursing).ab,id,ti. | 61 |
| 27 | (telepharmacy or tele-pharmacy).ab,id,ti. | 5 |
| 28 | (teleconsult$ or tele-consult$).ab,id,ti. | 176 |
| 29 | (telerehabilitation or tele-rehabilitation).ab,id,ti. | 200 |
| 30 | (telehealth or tele-health or telecare or tele-care).ab,id,ti. | 1696 |
| 31 | (telediagn$ or tele-diagn$).ab,id,ti. | 17 |
| 32 | (telemonitor$ or tele-monitor$).ab,id,ti. | 183 |
| 33 | (teleradio$ or tele-radio$).ab,id,ti. | 43 |
| 34 | (telepathology or tele-pathology).ab,id,ti. | 14 |
| 35 | (telepsychiatry or tele-psychiatry).ab,id,ti. | 469 |
| 36 | (telecardiology or tele-cardiology).ab,id,ti. | 14 |
| 37 | (teledermatology or tele-dermatology).ab,id,ti. | 43 |
| 38 | (teletherapy or tele-therapy).ab,id,ti. | 80 |
| 39 | "remote consultation".ab,id,ti. | 31 |
| 40 | (telecommunication$ or tele-communication$).ab,id,ti. | 2040 |
| 41 | ("video conferenc$" or videoconferenc$ or video-conferenc$).ab,id,ti. | 1769 |
| 42 | telephone$.ab,id,ti. | 23483 |
| 43 | (teleconferenc$ or tele-conferenc$).ab,id,ti. | 444 |
| 44 | telecommunications media/ or telephone systems/ or teleconferencing/ or telemedicine/ | 8590 |
| 45 | (mHealth or m-Health or "mobile Health").ab,id,ti. | 1263 |
| 46 | ("mobile telephone$" or "mobile phone$").ab,id,ti. | 3447 |
| 47 | ("cell$ phone$" or cellphone$ or cell-phone$).ab,id,ti. | 2082 |
| 48 | ("smart phone$" or "smartphone$" or smart-phone$).ab,id,ti. | 3711 |
| 49 | ("handheld computer$" or "hand-held computer$").ab,id,ti. | 389 |
| 50 | ("palmtop computer$" or "palm-top computer$").ab,id,ti. | 133 |
| 51 | ("tablet computer$" or "tablet PC").ab,id,ti. | 400 |
| 52 | ("personal digital assistant$" or PDA or PDAs).ab,id,ti. | 1065 |
| 53 | "mobile app$".ab,id,ti. | 1257 |
| 54 | "medical app$".ab,id,ti. | 1120 |
| 55 | "health app$".ab,id,ti. | 2276 |
| 56 | "short messag$ service$".ab,id,ti. | 426 |
| 57 | ("text messag$" or text-messag$).ab,id,ti. | 2113 |
| 58 | ("multimedia messag$ servic$" or "multi-media messag$ servic$").ab,id,ti. | 19 |
| 59 | ("mp3 player$" or mp3-player$).ab,id,ti. | 143 |
| 60 | ("handheld device$" or "hand-held device$" or "mobile device$").ab,id,ti. | 2292 |
| 61 | ("assist$ living device$" or wearable$ or fitbit or "digital health").ab,id,ti. | 1282 |
| 62 | mobile devices/ or cellular phones/ or text messaging/ | 6981 |
| 63 | "software app$".ab,id,ti. | 550 |
| 64 | "clinical decision support".ab,id,ti. | 400 |
| 65 | "computer-assisted decision$".ab,id,ti. | 21 |
| 66 | "computer-assisted diagnos#s".ab,id,ti. | 51 |
| 67 | "computer-assisted therapy".ab,id,ti. | 88 |
| 68 | (CDSS or CDS).ab,id,ti. | 1343 |
| 69 | "computer-assisted surgery".ab,id,ti. | 7 |
| 70 | "decision support system$".ab,id,ti. | 1916 |
| 71 | "computer app$".ab,id,ti. | 1134 |
| 72 | "computer software$".ab,id,ti. | 1642 |
| 73 | "computer-assisted instruction$".ab,id,ti. | 1802 |
| 74 | computer applications/ or computer assisted diagnosis/ or computer assisted instruction/ or computer assisted testing/ or computer assisted therapy/ | 31947 |
| 75 | decision support systems/ or computer software/ | 13170 |
| 76 | "health$ information system$".ab,id,ti. | 275 |
| 77 | "medical information system$".ab,id,ti. | 62 |
| 78 | "laboratory information system$".ab,id,ti. | 13 |
| 79 | "pharmacy information system$".ab,id,ti. | 5 |
| 80 | ("electronic health record$" or EHR).ab,id,ti. | 1682 |
| 81 | ("electronic medical record$" or EMR or "electronic patient record$" or "patient health record$" or PHR or "patient portal").ab,id,ti. | 2661 |
| 82 | "clinical information system$".ab,id,ti. | 114 |
| 83 | "computeri#ed medical record$".ab,id,ti. | 74 |
| 84 | "computeri#ed health record$".ab,id,ti. | 4 |
| 85 | skype.ab,id,ti. | 428 |
| 86 | "hospital information system$".ab,id,ti. | 85 |
| 87 | information systems/ | 5329 |
| 88 | medical records/ | 2854 |
| 89 | internet.ab,id,ti. | 38769 |
| 90 | "world wide web".ab,id,ti. | 1614 |
| 91 | ("social media" or "social network$").ab,id,ti. | 33596 |
| 92 | blog$.ab,id,ti. | 3113 |
| 93 | facebook.ab,id,ti. | 4831 |
| 94 | (twitter or tweet$).ab,id,ti. | 2679 |
| 95 | whatsapp.ab,id,ti. | 110 |
| 96 | instagram.ab,id,ti. | 359 |
| 97 | linkedin.ab,id,ti. | 271 |
| 98 | (e-mail or email or "electronic mail").ab,id,ti. | 7686 |
| 99 | computer mediated communication/ or electronic communication/ or blog/ or online community/ or online social networks/ or online therapy/ or social media/ | 23658 |
| 100 | internet/ or websites/ | 31599 |
| 101 | ("electronic prescri$" or eprescri$ or e-prescri$).ab,id,ti. | 105 |
| 102 | ("computer$ physician order entry" or "electronic order entry").ab,id,ti. | 54 |
| 103 | (web or website$).ab,id,ti. | 42674 |
| 104 | "medicine 2.0".ab,id,ti. | 21 |
| 105 | "health 2.0".ab,id,ti. | 79 |
| 106 | 23 or 24 or 25 or 26 or 27 or 28 or 29 or 30 or 31 or 32 or 33 or 34 or 35 or 36 or 37 or 38 or 39 or 40 or 41 or 42 or 43 or 44 or 45 or 46 or 47 or 48 or 49 or 50 or 51 or 52 or 53 or 54 or 55 or 56 or 57 or 58 or 59 or 60 or 61 or 62 or 63 or 64 or 65 or 66 or 67 or 68 or 69 or 70 or 71 or 72 or 73 or 74 or 75 or 76 or 77 or 78 or 79 or 80 or 81 or 82 or 83 or 84 or 85 or 86 or 87 or 88 or 89 or 90 or 91 or 92 or 93 or 94 or 95 or 96 or 97 or 98 or 99 or 100 or 101 or 102 or 103 or 104 or 105 | 204984 |
| 107 | 12 or 13 or 14 or 15 or 16 or 17 or 18 or 19 or 20 or 21 or 22 | 1926514 |
| 108 | 11 and 106 and 107 | 1107 |

Database(s): **Ovid MEDLINE(R) ALL**1946 to October 22, 2019

| **#** | **Searches** | **Results** |
| --- | --- | --- |
| 1 | (information adj1 quality).ti,ab,kw. | 2053 |
| 2 | (data adj1 quality).ti,ab,kw. | 11608 |
| 3 | (information adj1 reliab*).ti,ab,kw. | 3982 |
| 4 | (data adj1 reliab*).ti,ab,kw. | 5310 |
| 5 | (information adj1 valid*).ti,ab,kw. | 1036 |
| 6 | (data adj1 valid*).ti,ab,kw. | 6674 |
| 7 | (information adj1 audit*).ti,ab,kw. | 1692 |
| 8 | (data adj1 audit*).ti,ab,kw. | 922 |
| 9 | data accuracy/ or (data adj1 accura*).ti,ab,kw. or (information adj1 accura*).ti,ab,kw. | 10861 |
| 10 | framework*.ti,ab,kw. | 254226 |
| 11 | dimension*.ti,ab,kw. | 544302 |
| 12 | scale*.ti,ab,kw. | 772185 |
| 13 | indicator*.ti,ab,kw. | 264171 |
| 14 | guideline*.ti,ab,kw. | 327002 |
| 15 | metric*.ti,ab,kw. | 55858 |
| 16 | measur*.ti,ab,kw. | 3332908 |
| 17 | criteri*.ti,ab,kw. | 612815 |
| 18 | model*.ti,ab,kw. | 2721661 |
| 19 | standard*.ti,ab,kw. | 1200229 |
| 20 | Models, Theoretical/ | 146181 |
| 21 | (ehealth or e-health or "electronic health").ti,ab,kw. | 19309 |
| 22 | telemedicine/ or telepathology/ or teleradiology/ or telerehabilitation/ | 22733 |
| 23 | (telemedicine or tele-medicine).ti,ab,kw. | 11416 |
| 24 | (teleradio* or tele-radio*).ti,ab,kw. | 1638 |
| 25 | (telepathology or tele-pathology).ti,ab,kw. | 688 |
| 26 | (telerehabilitation or tele-rehabilitation).ti,ab,kw. | 808 |
| 27 | (teleconsult* or tele-consult*).ti,ab,kw. | 1222 |
| 28 | (telehealth or tele-health).ti,ab,kw. | 4318 |
| 29 | (teledermatology or tele-dermatology).ti,ab,kw. | 705 |
| 30 | (telecardiology or tele-cardiology).ti,ab,kw. | 230 |
| 31 | "remote consultation*".ti,ab,kw. | 326 |
| 32 | (telemonitor* or tele-monitor*).ti,ab,kw. | 1570 |
| 33 | (telediagn* or tele-diagn*).ti,ab,kw. | 220 |
| 34 | (telesurgery or tele-surgery).ti,ab,kw. | 286 |
| 35 | (telepsychiatry or tele-psychiatry).ti,ab,kw. | 559 |
| 36 | (teletherapy or tele-therapy).ti,ab,kw. | 1349 |
| 37 | Telenursing/ | 205 |
| 38 | (telenursing or tele-nursing).ti,ab,kw. | 198 |
| 39 | (telepharmacy or tele-pharmacy).ti,ab,kw. | 84 |
| 40 | (mHealth or m-Health or "mobile health").ti,ab,kw. | 5938 |
| 41 | ("digital health" or fitbit or wearable*).ti,ab,kw. | 11771 |
| 42 | computers, handheld/ | 3444 |
| 43 | ("hand-held computer*" or "handheld computer*" or "hand-held device*" or "handheld device*").ti,ab,kw. | 1746 |
| 44 | ("palmtop computer*" or "palm-top computer*").ti,ab,kw. | 132 |
| 45 | ("tablet computer*" or tablet PC).ti,ab,kw. | 893 |
| 46 | skype.ti,ab,kw. | 319 |
| 47 | ("personal digital assistant*" or PDA or PDAs).ti,ab,kw. | 11752 |
| 48 | "assist* living device*".ti,ab,kw. | 1 |
| 49 | telecommunications/ or telephone/ or videoconferencing/ | 17289 |
| 50 | (telecommunication* or tele-communication*).ti,ab,kw. | 4104 |
| 51 | (videoconferenc* or video-conferenc* or "video conferenc*").ti,ab,kw. | 2642 |
| 52 | (cellphone* or cell-phone* or "cell phone*" or "cellular phone*").ti,ab,kw. | 3540 |
| 53 | cell phones/ or smartphone/ or text messaging/ | 13094 |
| 54 | (smartphone* or smart-phone* or "smart phone*").ti,ab,kw. | 10313 |
| 55 | ("Short messag* service*" or SMS).ti,ab,kw. | 5802 |
| 56 | ("Multimedia messag* service*" or "Multi-media messag* service*" or MMS).ti,ab,kw. | 4580 |
| 57 | "mobile device*".ti,ab,kw. | 2836 |
| 58 | "software app*".ti,ab,kw. | 1903 |
| 59 | "mobile phone*".ti,ab,kw. | 7920 |
| 60 | "Mobile app*".ti,ab,kw. | 3914 |
| 61 | "medical app*".ti,ab,kw. | 10047 |
| 62 | "health app*".ti,ab,kw. | 5616 |
| 63 | ("text messag*" or text-messag*).ti,ab,kw. | 3736 |
| 64 | Mobile Applications/ | 4682 |
| 65 | MP3-Player/ | 181 |
| 66 | MP3 player*.ti,ab,kw. | 114 |
| 67 | MP3-player*.ti,ab,kw. | 114 |
| 68 | internet/ or blogging/ or social media/ | 75832 |
| 69 | (internet or web or website*).ti,ab,kw. | 159917 |
| 70 | blog*.ti,ab,kw. | 1706 |
| 71 | social media.ti,ab,kw. | 9496 |
| 72 | facebook.ti,ab,kw. | 3131 |
| 73 | (twitter or tweet*).ti,ab,kw. | 3740 |
| 74 | Whatsapp.ti,ab,kw. | 304 |
| 75 | Instagram.ti,ab,kw. | 360 |
| 76 | LinkedIn.ti,ab,kw. | 140 |
| 77 | health information systems/ or clinical laboratory information systems/ or clinical pharmacy information systems/ or hospital information systems/ | 15020 |
| 78 | Health Information system*.ti,ab,kw. | 3381 |
| 79 | ("Medical information system*" or "Clinical information system*").ti,ab,kw. | 2426 |
| 80 | "laboratory information system*".ti,ab,kw. | 957 |
| 81 | "pharmacy information system*".ti,ab,kw. | 96 |
| 82 | "Hospital Information system*".ti,ab,kw. | 2559 |
| 83 | "Nursing Information System*".ti,ab,kw. | 248 |
| 84 | "Decision Support".ti,ab,kw. | 13567 |
| 85 | electronic health records/ or medical records systems, computerized/ | 36228 |
| 86 | ("electronic health record*" or "electronic medical record*" or "electronic patient record*" or "patient health record*" or EHR or EMR or PHR or "computeri#ed health record*" or "computeri#ed medical record*" or "patient portal").ti,ab,kw. | 37353 |
| 87 | "Electronic Medical Record*".ti,ab,kw. | 14857 |
| 88 | Electronic Prescribing/ | 976 |
| 89 | "electronic prescri*".ti,ab,kw. | 1047 |
| 90 | hospital information systems/ or ambulatory care information systems/ or medical order entry systems/ or operating room information systems/ | 14383 |
| 91 | medical order entry systems/ or online systems/ or decision support systems, clinical/ | 17233 |
| 92 | decision making, computer-assisted/ or diagnosis, computer-assisted/ or image interpretation, computer-assisted/ or radiographic image interpretation, computer-assisted/ or therapy, computer-assisted/ or drug therapy, computer-assisted/ or radiotherapy, computer-assisted/ or surgery, computer-assisted/ | 105689 |
| 93 | ("computer* physician order entry" or "electronic order entry").ti,ab,kw. | 915 |
| 94 | ("medicine 2.0" or "health 2.0").ti,ab,kw. | 196 |
| 95 | (email or e-mail or "electronic mail").ti,ab,kw. | 12668 |
| 96 | 1 or 2 or 3 or 4 or 5 or 6 or 7 or 8 or 9 | 42802 |
| 97 | 21 or 22 or 23 or 24 or 25 or 26 or 27 or 28 or 29 or 30 or 31 or 32 or 33 or 34 or 35 or 36 or 37 or 38 or 39 or 40 or 41 or 42 or 43 or 44 or 45 or 46 or 47 or 48 or 49 or 50 or 51 or 52 or 53 or 54 or 55 or 56 or 57 or 58 or 59 or 60 or 61 or 62 or 63 or 64 or 65 or 66 or 67 or 68 or 69 or 70 or 71 or 72 or 73 or 74 or 75 or 76 or 77 or 78 or 79 or 80 or 81 or 82 or 83 or 84 or 85 or 86 or 87 or 88 or 89 or 90 or 91 or 92 or 93 or 94 or 95 | 506048 |
| 98 | 10 or 11 or 12 or 13 or 14 or 15 or 16 or 17 or 18 or 19 or 20 | 7763544 |
| 99 | 96 and 97 and 98 | 3543 |

Interface - EBSCOhost Research Databases
Database – CINAHL

Search modes - Boolean/Phrase

Friday, October 25, 2019 8:21:28 AM

| # | Query | Results |
| --- | --- | --- |
| S68 | S65 AND S66 AND S67 | 1,744 |
| S67 | S48 OR S49 OR S50 OR S51 OR S52 | 13,857 |
| S66 | S53 OR S54 OR S55 OR S56 OR S57 OR S58 OR S59 OR S60 OR S61 OR S62 OR S63 OR S64 | 1,460,243 |
| S65 | S1 OR S2 OR S3 OR S4 OR S5 OR S6 OR S7 OR S8 OR S9 OR S10 OR S11 OR S12 OR S13 OR S14 OR S15 OR S16 OR S17 OR S18 OR S19 OR S20 OR S21 OR S22 OR S23 OR S24 OR S25 OR S26 OR S27 OR S28 OR S29 OR S30 OR S31 OR S32 OR S33 OR S34 OR S35 OR S36 OR S37 OR S38 OR S39 OR S40 OR S41 OR S42 OR S43 OR S44 OR S45 OR S46 OR S47 | 275,283 |
| S64 | (MH "Models, Theoretical") | 41,146 |
| S63 | (MH "Conceptual Framework") | 39,808 |
| S62 | TI standard* OR AB standard* | 263,577 |
| S61 | TI criteri* OR AB criteri* | 152,590 |
| S60 | TI measur* OR AB measur* | 622,527 |
| S59 | TI metric* OR AB metric* | 12,879 |
| S58 | TI guideline* OR AB guideline* | 124,798 |
| S57 | TI indicator* OR AB indicator* | 54,469 |
| S56 | TI dimension* OR AB dimension* | 67,532 |
| S55 | TI scale* OR AB Scale* | 194,059 |
| S54 | TI model* OR AB model* | 427,257 |
| S53 | TI framework* OR AB framework* | 73,896 |
| S52 | TI information N1 audit* OR AB information N1 audit* OR TI data N1 audit* OR AB data N1 audit* | 1,020 |
| S51 | TI information N1 reliab* OR AB information N1 reliab* OR TI data N1 reliab* OR AB data N1 reliab* | 3,070 |
| S50 | TI information N1 valid* OR AB information N1 valid* OR TI information N1 valid* OR AB information N1 valid* | 575 |
| S49 | TI information N1 accura* OR AB information N1 accura* OR TI information N1 accura* OR AB information N1 accura* | 2,291 |
| S48 | TI information N1 quality OR AB information N1 quality OR TI data N1 quality OR AB data N1 quality | 7,214 |
| S47 | (MH "Blogs") | 2,935 |
| S46 | (MH "Social Media") | 11,694 |
| S45 | (MH "Internet") | 44,106 |
| S44 | TI ( internet or "social media" or "social network*" or web or website or facebook or email or e-mail or "electronic mail" or linkedin or whatsapp or skype or instagram or blog* ) OR AB ( internet or "social media" or "social network*" or web or website or facebook or email or e-mail or "electronic mail" or linkedin or whatsapp or skype or instagram or blog* ) | 99,829 |
| S43 | (MH "Electronic Order Entry") | 2,926 |
| S42 | TI ( "electronic order entry" or "computeri#ed order entry" or "computeri#ed physician order entry" or eprescri* or e-prescri* ) OR AB ( "electronic order entry" or "computeri#ed order entry" or "computeri#ed physician order entry" or eprescri* or e-prescri* ) | 1,068 |
| S41 | TI ( "computeri#ed medical record*" or "computeri#ed patient record*" or "computeri#ed health record*" ) OR AB ( "computeri#ed medical record*" or "computeri#ed patient record*" or "computeri#ed health record*" ) | 412 |
| S40 | (MH "Computerized Patient Record") | 481 |
| S39 | TI ( "electronic health record*" or "electronic medical record*" or "EMR" or "EHR" or "patient health record*" or "PHR" or "electronic patient record*" or "patient portal" ) OR AB ( "electronic health record*" or "electronic medical record*" or "EMR" or "EHR" or "patient health record*" or "PHR" or "electronic patient record*" or "patient portal" ) | 20,249 |
| S38 | (MH "Nursing Information Systems") OR (MH "Radiology Information Systems") | 2,540 |
| S37 | (MH "Clinical Pharmacy Information Systems") | 1,041 |
| S36 | (MH "Clinical Laboratory Information Systems") | 882 |
| S35 | (MH "Health Information Systems") OR (MH "Home Health Care Information Systems") OR (MH "Hospital Information Systems") OR (MH "Operating Room Information Systems") OR (MH "Clinical Information Systems") OR (MH "Patient Record Systems") OR (MH "Clinical Pharmacy Information Systems") OR (MH "Emergency Service Information Systems") | 21,042 |
| S34 | TI ( "health information system*" OR "healthcare information system*" OR "medical information system*" OR "laboratory information system*" OR "pharmacy information system*" OR "nursing information system*" OR "clinical information system*" OR "hospital information system*" ) AND AB ( "health information system*" OR "healthcare information system*" OR "medical information system*" OR "laboratory information system*" OR "pharmacy information system*" OR "nursing information system*" OR "clinical information system*" OR "hospital information system*" ) | 477 |
| S33 | (MH "Diagnosis, Computer Assisted") OR (MH "Surgery, Computer-Assisted") OR (MH "Therapy, Computer Assisted") OR (MH "Radiographic Image Interpretation, Computer-Assisted") OR (MH "Drug Therapy, Computer Assisted") OR (MH "Radiotherapy, Computer-Assisted") OR (MH "Image Interpretation, Computer Assisted") OR (MH "Decision Making, Computer Assisted") | 27,834 |
| S32 | (MH "Decision Support Systems, Clinical") | 4,524 |
| S31 | TI ( "computer app*" or "computer software*" ) OR AB ( "computer app*" or "computer software*" ) | 1,643 |
| S30 | TI ( "software app*" or "clinical decision support" or "decision support system*" or "computer-assisted decision*" or "computer-assisted diagnos#s" or "computer-assisted therapy" or "CDS" or "CDSS" or "computer-assisted surgery" or "computer-assisted radiology" ) OR AB ( "software app*" or "clinical decision support" or "decision support system*" or "computer-assisted decision*" or "computer-assisted diagnos#s" or "computer-assisted therapy" or "CDS" or "CDSS" or "computer-assisted surgery" or "computer-assisted radiology" ) | 5,024 |
| S29 | (MH "Smartphone") OR (MH "Text Messaging") OR (MH "Mobile Applications") OR (MH "Computers, Hand-Held") OR (MH "Cellular Phone") | 14,471 |
| S28 | TI ( "mp3 player*" or "mp3-player*" ) OR AB ( "mp3 player*" or "mp3-player*" ) | 81 |
| S27 | TI ( "short messag* service*" or "text messag*" or text-messag* or "multimedia messag* service*" or "multi-media messag* service*" ) OR AB ( "short messag* service*" or "text messag*" or text-messag* or "multimedia messag* service*" or "multi-media messag* service*" ) | 2,643 |
| S26 | TI ( "mobile app*" or "medical app*" or "health app*" ) OR AB ( "mobile app*" or "medical app*" or "health app*" ) | 6,084 |
| S25 | TI ( "personal digital assistant*" or "PDA" or "PDAs" ) OR AB ( "personal digital assistant*" or "PDA" or "PDAs" ) | 2,076 |
| S24 | TI ( "palmtop computer*" or "palm-top computer*" or "tablet computer*" or "tablet PC" ) OR AB ( "palmtop computer*" or "palm-top computer*" or "tablet computer*" or "tablet PC" ) | 523 |
| S23 | TI ( "handheld computer*" or "hand-held computer*" or "handheld device*" or "hand-held device*" ) OR AB ( "handheld computer*" or "hand-held computer*" or "handheld device*" or "hand-held device*" ) | 705 |
| S22 | TI ( smartphone* or smart-phone* or "smart phone*" ) OR AB ( smartphone* or smart-phone* or "smart phone*" ) | 4,827 |
| S21 | TI ( "cell* phone*" or cellphone* or cell-phone* ) OR AB ( "cell* phone*" or cellphone* or cell-phone* ) | 1,648 |
| S20 | TI ( "mobile phone*" or "mobile telephone*" or "mobile device*" ) OR AB ( "mobile phone*" or "mobile telephone*" or "mobile device*" ) | 4,282 |
| S19 | TI ( mhealth or "mobile health" or m-health ) OR AB ( mhealth or "mobile health" or m-health ) | 2,262 |
| S18 | (MH "Remote Consultation") OR (MH "Telephone Consultation (Iowa NIC)") | 1,742 |
| S17 | (MH "Teleconferencing") | 1,319 |
| S16 | (MH "Telehealth") | 6,825 |
| S15 | (MH "Telepathology") | 142 |
| S14 | (MH "Telecommunications") | 2,145 |
| S13 | (MH "Telephone") | 15,379 |
| S12 | (MH "Teleradiology") | 463 |
| S11 | (MH "Telepsychiatry") | 300 |
| S10 | (MH "Telenursing") | 1,926 |
| S9 | (MH "Telemedicine") OR (MH "Telepsychiatry") OR (MH "Telehealth") | 16,471 |
| S8 | TI ( teleconferenc* or tele-conferenc* ) OR AB ( teleconferenc* or tele-conferenc* ) OR TI ( videoconferenc* or video-conferenc* or "video conferenc*" ) OR AB ( videoconferenc* or video-conferenc* or "video conferenc*" ) | 1,985 |
| S7 | TI ( tele-phone* or telephone* or telecommunication* or tele-communication* ) OR AB ( tele-phone* or telephone* or telecommunication* or tele-communication* ) | 27,326 |
| S6 | TI "remote consultation" OR AB "remote consultation" | 46 |
| S5 | TI ( telepathology or telecardiology or telepsychiatry or teleradio* or teledermatology ) OR AB ( telepathology or telecardiology or telepsychiatry or teleradio* or teledermatology ) OR TI ( tele-pathology or tele-cardiology or tele-psychiatry or tele-radio* or tele-dermatology ) OR AB ( tele-pathology or tele-cardiology or tele-psychiatry or tele-radio* or tele-dermatology ) | 1,090 |
| S4 | TI ( telenursing or telepharmacy or telerehabilitation or tele-nursing or tele-pharmacy or tele-rehabilitation ) OR AB ( telenursing or telepharmacy or telerehabilitation or tele-nursing or tele-pharmacy or tele-rehabilitation ) | 647 |
| S3 | TI ( teleconsult* or telediag* or telemonitor* or tele-consult* or tele-diag* or tele-monitor* or teletherapy or tele-therapy ) OR AB ( teleconsult* or telediag* or telemonitor* or tele-consult* or tele-diag* or tele-monitor* or teletherapy or tele-therapy ) | 1,214 |
| S2 | TI ( telemedicine or telehealth or telecare or tele-medicine or tele-health or tele-care ) OR AB ( telemedicine or telehealth or telecare or tele-medicine or tele-health or tele-care ) | 7,614 |
| S1 | TI ( "electronic health" or ehealth or e-health ) OR AB ( "electronic health" or ehealth or e-health ) | 12,120 |

**Databases: ProQuest Dissertations & Theses Global**

**25 October 2019 12:52**

| Set# | Searched for | Results |
| --- | --- | --- |
| S2 | ti(framework* OR model* OR scale* OR dimension* OR indicator* OR guideline* OR standard* OR measur* OR metric* OR criteri*) OR ab(framework* OR model* OR scale* OR dimension* OR indicator* OR guideline* OR standard* OR measur* OR metric* OR criteri*) | 2001228 |
| S3 | (ti(information NEAR/1 quality) OR ab(information NEAR/1 quality) OR ti(data NEAR/1 quality) OR ab(data NEAR/1 quality)) OR (ti(information NEAR/1 accura*) OR ab(information NEAR/1 accura*) OR ti(data NEAR/1 accura*) OR ab(data NEAR/1 accura*)) OR (ti(information NEAR/1 reliab*) OR ab(information NEAR/1 reliab*) OR ti(data NEAR/1 reliab*) OR ab(data NEAR/1 reliab*)) OR (ti(information NEAR/1 valid*) OR ab(information NEAR/1 valid*) OR ti(data NEAR/1 valid*) OR ab(data NEAR/1 valid*)) | 23135 |
| S4 | (ti("health information system*" OR "healthcare information system*" OR "medical information system*" OR "laboratory information system*" OR "pharmacy information system*" OR "nursing information system*" OR "clinical information system*" OR "hospital information system*") OR ab("health information system*" OR "healthcare information system*" OR "medical information system*" OR "laboratory information system*" OR "pharmacy information system*" OR "nursing information system*" OR "clinical information system*" OR "hospital information system*")) OR (ti("electronic health record*" OR "electronic medical record*" OR "EMR" OR "EHR" OR "patient health record*" OR "PHR" OR "electronic patient record*" OR "patient portal") OR ab("electronic health record*" OR "electronic medical record*" OR "EMR" OR "EHR" OR "patient health record*" OR "PHR" OR "electronic patient record*" OR "patient portal")) OR (ti("clinical decision support" OR "decision support system*" OR "computer-assisted decision*" OR "computer-assisted diagnos#s" OR "computer-assisted therapy" OR "CDS" OR "CDSS" OR "computer-assisted surgery" OR "computer-assisted radio*") OR ab("clinical decision support" OR "decision support system*" OR "computer-assisted decision*" OR "computer-assisted diagnos#s" OR "computer-assisted therapy" OR "CDS" OR "CDSS" OR "computer-assisted surgery" OR "computer-assisted radio*")) OR (ti("electronic order entry" OR "computerized order entry" OR "computerized physician order entry" OR eprescri* OR e-prescri* OR "computerised order entry" OR "computerised physician order entry") OR ab("electronic order entry" OR "computerized order entry" OR "computerized physician order entry" OR eprescri* OR e-prescri* OR "computerised order entry" OR "computerised physician order entry")) OR (ti("computerized medical record*" OR "computerized patient record*" OR "computerized health record*") OR ab("computerized medical record*" OR "computerized patient record*" OR "computerized health record*")) OR (ti(medical NEAR/4 app*) OR ab(medical NEAR/4 app*) OR ti(health NEAR/4 app*) OR ab(health NEAR/4 app*)) OR (ti(telepathology OR telecardiology OR telepsychiatry OR teleradio* OR teledermatology) OR ab(telepathology OR telecardiology OR telepsychiatry OR teleradio* OR teledermatology)) OR (ti(telemedicine OR telehealth OR telecare OR tele-medicine OR tele-health OR tele-care) OR ab(telemedicine OR telehealth OR telecare OR tele-medicine OR tele-health OR tele-care)) OR (ab(teleconsult* OR telediag* OR telemonitor* OR tele-consult* OR tele-diag* OR tele-monitor* OR teletherapy OR tele-therapy) OR ti(teleconsult* OR telediag* OR telemonitor* OR tele-consult* OR tele-diag* OR tele-monitor* OR teletherapy OR tele-therapy)) OR (ab(telenursing OR telepharmacy OR telerehabilitation OR tele-nursing OR tele-pharmacy OR tele-rehabilitation) OR ti(telenursing OR telepharmacy OR telerehabilitation OR tele-nursing OR tele-pharmacy OR tele-rehabilitation)) OR (ti("remote consultation") OR ab("remote consultation")) OR (ti(tele-pathology OR tele-cardiology OR tele-psychiatry OR tele-radio* OR tele-dermatology) OR ab(tele-pathology OR tele-cardiology OR tele-psychiatry OR tele-radio* OR tele-dermatology)) OR (ti("electronic health" OR ehealth OR e-health) OR ab("electronic health" OR ehealth OR e-health)) | 18163 |
| S5 | (ti(framework* OR model* OR scale* OR dimension* OR indicator* OR guideline* OR standard* OR measur* OR metric* OR criteri*) OR ab(framework* OR model* OR scale* OR dimension* OR indicator* OR guideline* OR standard* OR measur* OR metric* OR criteri*)) AND ((ti(information NEAR/1 quality) OR ab(information NEAR/1 quality) OR ti(data NEAR/1 quality) OR ab(data NEAR/1 quality)) OR (ti(information NEAR/1 accura*) OR ab(information NEAR/1 accura*) OR ti(data NEAR/1 accura*) OR ab(data NEAR/1 accura*)) OR (ti(information NEAR/1 reliab*) OR ab(information NEAR/1 reliab*) OR ti(data NEAR/1 reliab*) OR ab(data NEAR/1 reliab*)) OR (ti(information NEAR/1 valid*) OR ab(information NEAR/1 valid*) OR ti(data NEAR/1 valid*) OR ab(data NEAR/1 valid*))) AND ((ti("health information system*" OR "healthcare information system*" OR "medical information system*" OR "laboratory information system*" OR "pharmacy information system*" OR "nursing information system*" OR "clinical information system*" OR "hospital information system*") OR ab("health information system*" OR "healthcare information system*" OR "medical information system*" OR "laboratory information system*" OR "pharmacy information system*" OR "nursing information system*" OR "clinical information system*" OR "hospital information system*")) OR (ti("electronic health record*" OR "electronic medical record*" OR "EMR" OR "EHR" OR "patient health record*" OR "PHR" OR "electronic patient record*" OR "patient portal") OR ab("electronic health record*" OR "electronic medical record*" OR "EMR" OR "EHR" OR "patient health record*" OR "PHR" OR "electronic patient record*" OR "patient portal")) OR (ti("clinical decision support" OR "decision support system*" OR "computer-assisted decision*" OR "computer-assisted diagnos#s" OR "computer-assisted therapy" OR "CDS" OR "CDSS" OR "computer-assisted surgery" OR "computer-assisted radio*") OR ab("clinical decision support" OR "decision support system*" OR "computer-assisted decision*" OR "computer-assisted diagnos#s" OR "computer-assisted therapy" OR "CDS" OR "CDSS" OR "computer-assisted surgery" OR "computer-assisted radio*")) OR (ti("electronic order entry" OR "computerized order entry" OR "computerized physician order entry" OR eprescri* OR e-prescri* OR "computerised order entry" OR "computerised physician order entry") OR ab("electronic order entry" OR "computerized order entry" OR "computerized physician order entry" OR eprescri* OR e-prescri* OR "computerised order entry" OR "computerised physician order entry")) OR (ti("computerized medical record*" OR "computerized patient record*" OR "computerized health record*") OR ab("computerized medical record*" OR "computerized patient record*" OR "computerized health record*")) OR (ti(medical NEAR/4 app*) OR ab(medical NEAR/4 app*) OR ti(health NEAR/4 app*) OR ab(health NEAR/4 app*)) OR (ti(telepathology OR telecardiology OR telepsychiatry OR teleradio* OR teledermatology) OR ab(telepathology OR telecardiology OR telepsychiatry OR teleradio* OR teledermatology)) OR (ti(telemedicine OR telehealth OR telecare OR tele-medicine OR tele-health OR tele-care) OR ab(telemedicine OR telehealth OR telecare OR tele-medicine OR tele-health OR tele-care)) OR (ab(teleconsult* OR telediag* OR telemonitor* OR tele-consult* OR tele-diag* OR tele-monitor* OR teletherapy OR tele-therapy) OR ti(teleconsult* OR telediag* OR telemonitor* OR tele-consult* OR tele-diag* OR tele-monitor* OR teletherapy OR tele-therapy)) OR (ab(telenursing OR telepharmacy OR telerehabilitation OR tele-nursing OR tele-pharmacy OR tele-rehabilitation) OR ti(telenursing OR telepharmacy OR telerehabilitation OR tele-nursing OR tele-pharmacy OR tele-rehabilitation)) OR (ti("remote consultation") OR ab("remote consultation")) OR (ti(tele-pathology OR tele-cardiology OR tele-psychiatry OR tele-radio* OR tele-dermatology) OR ab(tele-pathology OR tele-cardiology OR tele-psychiatry OR tele-radio* OR tele-dermatology)) OR (ti("electronic health" OR ehealth OR e-health) OR ab("electronic health" OR ehealth OR e-health))) | 334 |

**Database: Scopus**

| **History Count** | **Search Terms** | **Results** |
| --- | --- | --- |
| 12 | ( ( TITLE-ABS-KEY ( information  W/1  quality )  OR  TITLE-ABS-KEY ( data  W/1  quality )  OR  TITLE-ABS-KEY ( information  W/1  valid* )  OR  TITLE-ABS-KEY ( data  W/1  valid* )  OR  TITLE-ABS-KEY ( data  W/1  reliab* )  OR  TITLE-ABS-KEY ( information  W/1  reliab* )  OR  TITLE-ABS-KEY ( data  W/1  accura* )  OR  TITLE-ABS-KEY ( information  W/1  accura* )  OR  TITLE-ABS-KEY ( data  W/1  audit* )  OR  TITLE-ABS-KEY ( information  W/1  audit* ) ) )  AND  ( ( TITLE-ABS-KEY ( framework* )  OR  TITLE-ABS-KEY ( model* )  OR  TITLE-ABS-KEY ( scale* )  OR  TITLE-ABS-KEY ( dimension* )  OR  TITLE-ABS-KEY ( indicator* )  OR  TITLE-ABS-KEY ( guideline* )  OR  TITLE-ABS-KEY ( metric* )  OR  TITLE-ABS-KEY ( measur* )  OR  TITLE-ABS-KEY ( criteri* )  OR  TITLE-ABS-KEY ( standard* ) ) )  AND  ( ( ( TITLE-ABS-KEY ( ehealth )  OR  TITLE-ABS-KEY ( e-health )  OR  TITLE-ABS-KEY ( "electronic health" )  OR  TITLE-ABS-KEY ( telemedicine )  OR  TITLE-ABS-KEY ( telemedicine )  OR  TITLE-ABS-KEY ( telenursing )  OR  TITLE-ABS-KEY ( tele-nursing )  OR  TITLE-ABS-KEY ( telepharmacy )  OR  TITLE-ABS-KEY ( tele-pharmacy )  OR  TITLE-ABS-KEY ( teleconsult* )  OR  TITLE-ABS-KEY ( tele-consult* )  OR  TITLE-ABS-KEY ( telerehabilitation )  OR  TITLE-ABS-KEY ( tele-rehabilitation )  OR  TITLE-ABS-KEY ( teleradio* )  OR  TITLE-ABS-KEY ( tele-radio* )  OR  TITLE-ABS-KEY ( telehealth )  OR  TITLE-ABS-KEY ( tele-health )  OR  TITLE-ABS-KEY ( telediagn* )  OR  TITLE-ABS-KEY ( tele-diagn* )  OR  TITLE-ABS-KEY ( telepathology )  OR  TITLE-ABS-KEY ( tele-pathology )  OR  TITLE-ABS-KEY ( telepsychiatry )  OR  TITLE-ABS-KEY ( tele-psychiatry )  OR  TITLE-ABS-KEY ( telecardiology )  OR  TITLE-ABS-KEY ( tele-cardiology )  OR  TITLE-ABS-KEY ( teledermatology )  OR  TITLE-ABS-KEY ( tele-dermatology )  OR  TITLE-ABS-KEY ( "remote consultation" )  OR  TITLE-ABS-KEY ( teletherapy )  OR  TITLE-ABS-KEY ( teletherapy )  OR  TITLE-ABS-KEY ( telecare )  OR  TITLE-ABS-KEY ( tele-care ) ) )  OR  ( ( TITLE-ABS-KEY ( "mobile health" )  OR  TITLE-ABS-KEY ( mhealth )  OR  TITLE-ABS-KEY ( m-health )  OR  TITLE-ABS-KEY ( "health W/4 app*" )  OR  TITLE-ABS-KEY ( "medical W/4 app*" ) ) )  OR  ( ( TITLE-ABS-KEY ( "clinical decision support" )  OR  TITLE-ABS-KEY ( "computer-assisted decision*" )  OR  TITLE-ABS-KEY ( "computer-assisted diagnosis" )  OR  TITLE-ABS-KEY ( "computer-assisted therapy" )  OR  TITLE-ABS-KEY ( "computer-assisted surgery" )  OR  TITLE-ABS-KEY ( "computer-assisted radiology" )  OR  TITLE-ABS-KEY ( "decision support system*" ) ) )  OR  ( ( TITLE-ABS-KEY ( "health information system*" )  OR  TITLE-ABS-KEY ( "medical information system*" )  OR  TITLE-ABS-KEY ( "healthcare information system*" )  OR  TITLE-ABS-KEY ( "clinical information system*" )  OR  TITLE-ABS-KEY ( "laboratory information system*" )  OR  TITLE-ABS-KEY ( "pharmacy information system*" )  OR  TITLE-ABS-KEY ( "nursing information system*" )  OR  TITLE-ABS-KEY ( "hospital information system*" )  OR  TITLE-ABS-KEY ( "operating room information system*" ) ) )  OR  ( ( TITLE-ABS-KEY ( "digital health" )  OR  TITLE-ABS-KEY ( wearable* )  OR  TITLE-ABS-KEY ( fitbit )  OR  TITLE-ABS-KEY ( "assistive living device*" )  OR  TITLE-ABS-KEY ( "assisted living device*" ) ) )  OR  ( ( TITLE-ABS-KEY ( epriscri* )  OR  TITLE-ABS-KEY ( "electronic order entry" )  OR  TITLE-ABS-KEY ( "computer physician order entry" )  OR  TITLE-ABS-KEY ( "computeri#ed physician order entry" )  OR  TITLE-ABS-KEY ( "electronic physician order entry" )  OR  TITLE-ABS-KEY ( e-prescr* ) ) )  OR  ( ( TITLE-ABS-KEY ( "web-based health service*" )  OR  TITLE-ABS-KEY ( "health 2.0" )  OR  TITLE-ABS-KEY ( "medicine 2.0" ) ) )  OR  ( ( TITLE-ABS-KEY ( "electronic health record*" )  OR  TITLE-ABS-KEY ( "electronic medical record*" )  OR  TITLE-ABS-KEY ( "electronic patient record*" )  OR  TITLE-ABS-KEY ( "computeri#ed health record*" )  OR  TITLE-ABS-KEY ( "computeri#ed medical record*" )  OR  TITLE-ABS-KEY ( "patient portal" )  OR  TITLE-ABS-KEY ( "patient health record*" )  OR  TITLE-ABS-KEY ( ehr )  OR  TITLE-ABS-KEY ( emr )  OR  TITLE-ABS-KEY ( phr ) ) ) )  ...View More | [5,461 document results](https://www.scopus.com/search/history/results.uri?origin=searchhistory&shid=15) |
| 11 | ( ( TITLE-ABS-KEY ( ehealth )  OR  TITLE-ABS-KEY ( e-health )  OR  TITLE-ABS-KEY ( "electronic health" )  OR  TITLE-ABS-KEY ( telemedicine )  OR  TITLE-ABS-KEY ( telemedicine )  OR  TITLE-ABS-KEY ( telenursing )  OR  TITLE-ABS-KEY ( tele-nursing )  OR  TITLE-ABS-KEY ( telepharmacy )  OR  TITLE-ABS-KEY ( tele-pharmacy )  OR  TITLE-ABS-KEY ( teleconsult* )  OR  TITLE-ABS-KEY ( tele-consult* )  OR  TITLE-ABS-KEY ( telerehabilitation )  OR  TITLE-ABS-KEY ( tele-rehabilitation )  OR  TITLE-ABS-KEY ( teleradio* )  OR  TITLE-ABS-KEY ( tele-radio* )  OR  TITLE-ABS-KEY ( telehealth )  OR  TITLE-ABS-KEY ( tele-health )  OR  TITLE-ABS-KEY ( telediagn* )  OR  TITLE-ABS-KEY ( tele-diagn* )  OR  TITLE-ABS-KEY ( telepathology )  OR  TITLE-ABS-KEY ( tele-pathology )  OR  TITLE-ABS-KEY ( telepsychiatry )  OR  TITLE-ABS-KEY ( tele-psychiatry )  OR  TITLE-ABS-KEY ( telecardiology )  OR  TITLE-ABS-KEY ( tele-cardiology )  OR  TITLE-ABS-KEY ( teledermatology )  OR  TITLE-ABS-KEY ( tele-dermatology )  OR  TITLE-ABS-KEY ( "remote consultation" )  OR  TITLE-ABS-KEY ( teletherapy )  OR  TITLE-ABS-KEY ( teletherapy )  OR  TITLE-ABS-KEY ( telecare )  OR  TITLE-ABS-KEY ( tele-care ) ) )  OR  ( ( TITLE-ABS-KEY ( "mobile health" )  OR  TITLE-ABS-KEY ( mhealth )  OR  TITLE-ABS-KEY ( m-health )  OR  TITLE-ABS-KEY ( "health W/4 app*" )  OR  TITLE-ABS-KEY ( "medical W/4 app*" ) ) )  OR  ( ( TITLE-ABS-KEY ( "clinical decision support" )  OR  TITLE-ABS-KEY ( "computer-assisted decision*" )  OR  TITLE-ABS-KEY ( "computer-assisted diagnosis" )  OR  TITLE-ABS-KEY ( "computer-assisted therapy" )  OR  TITLE-ABS-KEY ( "computer-assisted surgery" )  OR  TITLE-ABS-KEY ( "computer-assisted radiology" )  OR  TITLE-ABS-KEY ( "decision support system*" ) ) )  OR  ( ( TITLE-ABS-KEY ( "health information system*" )  OR  TITLE-ABS-KEY ( "medical information system*" )  OR  TITLE-ABS-KEY ( "healthcare information system*" )  OR  TITLE-ABS-KEY ( "clinical information system*" )  OR  TITLE-ABS-KEY ( "laboratory information system*" )  OR  TITLE-ABS-KEY ( "pharmacy information system*" )  OR  TITLE-ABS-KEY ( "nursing information system*" )  OR  TITLE-ABS-KEY ( "hospital information system*" )  OR  TITLE-ABS-KEY ( "operating room information system*" ) ) )  OR  ( ( TITLE-ABS-KEY ( "digital health" )  OR  TITLE-ABS-KEY ( wearable* )  OR  TITLE-ABS-KEY ( fitbit )  OR  TITLE-ABS-KEY ( "assistive living device*" )  OR  TITLE-ABS-KEY ( "assisted living device*" ) ) )  OR  ( ( TITLE-ABS-KEY ( epriscri* )  OR  TITLE-ABS-KEY ( "electronic order entry" )  OR  TITLE-ABS-KEY ( "computer physician order entry" )  OR  TITLE-ABS-KEY ( "computeri#ed physician order entry" )  OR  TITLE-ABS-KEY ( "electronic physician order entry" )  OR  TITLE-ABS-KEY ( e-prescr* ) ) )  OR  ( ( TITLE-ABS-KEY ( "web-based health service*" )  OR  TITLE-ABS-KEY ( "health 2.0" )  OR  TITLE-ABS-KEY ( "medicine 2.0" ) ) )  OR  ( ( TITLE-ABS-KEY ( "electronic health record*" )  OR  TITLE-ABS-KEY ( "electronic medical record*" )  OR  TITLE-ABS-KEY ( "electronic patient record*" )  OR  TITLE-ABS-KEY ( "computeri#ed health record*" )  OR  TITLE-ABS-KEY ( "computeri#ed medical record*" )  OR  TITLE-ABS-KEY ( "patient portal" )  OR  TITLE-ABS-KEY ( "patient health record*" )  OR  TITLE-ABS-KEY ( ehr )  OR  TITLE-ABS-KEY ( emr )  OR  TITLE-ABS-KEY ( phr ) ) )  ...View More | [404,296 document results](https://www.scopus.com/search/history/results.uri?origin=searchhistory&shid=13) |
| 10 | ( TITLE-ABS-KEY ( "electronic health record*" )  OR  TITLE-ABS-KEY ( "electronic medical record*" )  OR  TITLE-ABS-KEY ( "electronic patient record*" )  OR  TITLE-ABS-KEY ( "computeri#ed health record*" )  OR  TITLE-ABS-KEY ( "computeri#ed medical record*" )  OR  TITLE-ABS-KEY ( "patient portal" )  OR  TITLE-ABS-KEY ( "patient health record*" )  OR  TITLE-ABS-KEY ( ehr )  OR  TITLE-ABS-KEY ( emr )  OR  TITLE-ABS-KEY ( phr ) )  ...View More | [76,225 document results](https://www.scopus.com/search/history/results.uri?origin=searchhistory&shid=12) |
| 9 | ( TITLE-ABS-KEY ( "web-based health service*" )  OR  TITLE-ABS-KEY ( "health 2.0" )  OR  TITLE-ABS-KEY ( "medicine 2.0" ) ) | [245 document results](https://www.scopus.com/search/history/results.uri?origin=searchhistory&shid=11) |
| 8 | ( TITLE-ABS-KEY ( epriscri* )  OR  TITLE-ABS-KEY ( "electronic order entry" )  OR  TITLE-ABS-KEY ( "computer physician order entry" )  OR  TITLE-ABS-KEY ( "computeri#ed physician order entry" )  OR  TITLE-ABS-KEY ( "electronic physician order entry" )  OR  TITLE-ABS-KEY ( e-prescr* ) ) | [944 document results](https://www.scopus.com/search/history/results.uri?origin=searchhistory&shid=10) |
| 7 | ( TITLE-ABS-KEY ( "digital health" )  OR  TITLE-ABS-KEY ( wearable* )  OR  TITLE-ABS-KEY ( fitbit )  OR  TITLE-ABS-KEY ( "assistive living device*" )  OR  TITLE-ABS-KEY ( "assisted living device*" ) ) | [51,106 document results](https://www.scopus.com/search/history/results.uri?origin=searchhistory&shid=9) |
| 6 | ( TITLE-ABS-KEY ( "health information system*" )  OR  TITLE-ABS-KEY ( "medical information system*" )  OR  TITLE-ABS-KEY ( "healthcare information system*" )  OR  TITLE-ABS-KEY ( "clinical information system*" )  OR  TITLE-ABS-KEY ( "laboratory information system*" )  OR  TITLE-ABS-KEY ( "pharmacy information system*" )  OR  TITLE-ABS-KEY ( "nursing information system*" )  OR  TITLE-ABS-KEY ( "hospital information system*" )  OR  TITLE-ABS-KEY ( "operating room information system*" ) ) | [55,678 document results](https://www.scopus.com/search/history/results.uri?origin=searchhistory&shid=7) |
| 5 | ( TITLE-ABS-KEY ( "clinical decision support" )  OR  TITLE-ABS-KEY ( "computer-assisted decision*" )  OR  TITLE-ABS-KEY ( "computer-assisted diagnosis" )  OR  TITLE-ABS-KEY ( "computer-assisted therapy" )  OR  TITLE-ABS-KEY ( "computer-assisted surgery" )  OR  TITLE-ABS-KEY ( "computer-assisted radiology" )  OR  TITLE-ABS-KEY ( "decision support system*" ) ) | [167,343 document results](https://www.scopus.com/search/history/results.uri?origin=searchhistory&shid=6) |
| 4 | ( TITLE-ABS-KEY ( "mobile health" )  OR  TITLE-ABS-KEY ( mhealth )  OR  TITLE-ABS-KEY ( m-health )  OR  TITLE-ABS-KEY ( "health W/4 app*" )  OR  TITLE-ABS-KEY ( "medical W/4 app*" ) ) | [15,722 document results](https://www.scopus.com/search/history/results.uri?origin=searchhistory&shid=5) |
| 3 | ( TITLE-ABS-KEY ( ehealth )  OR  TITLE-ABS-KEY ( e-health )  OR  TITLE-ABS-KEY ( "electronic health" )  OR  TITLE-ABS-KEY ( telemedicine )  OR  TITLE-ABS-KEY ( telemedicine )  OR  TITLE-ABS-KEY ( telenursing )  OR  TITLE-ABS-KEY ( tele-nursing )  OR  TITLE-ABS-KEY ( telepharmacy )  OR  TITLE-ABS-KEY ( tele-pharmacy )  OR  TITLE-ABS-KEY ( teleconsult* )  OR  TITLE-ABS-KEY ( tele-consult* )  OR  TITLE-ABS-KEY ( telerehabilitation )  OR  TITLE-ABS-KEY ( tele-rehabilitation )  OR  TITLE-ABS-KEY ( teleradio* )  OR  TITLE-ABS-KEY ( tele-radio* )  OR  TITLE-ABS-KEY ( telehealth )  OR  TITLE-ABS-KEY ( tele-health )  OR  TITLE-ABS-KEY ( telediagn* )  OR  TITLE-ABS-KEY ( tele-diagn* )  OR  TITLE-ABS-KEY ( telepathology )  OR  TITLE-ABS-KEY ( tele-pathology )  OR  TITLE-ABS-KEY ( telepsychiatry )  OR  TITLE-ABS-KEY ( tele-psychiatry )  OR  TITLE-ABS-KEY ( telecardiology )  OR  TITLE-ABS-KEY ( tele-cardiology )  OR  TITLE-ABS-KEY ( teledermatology )  OR  TITLE-ABS-KEY ( tele-dermatology )  OR  TITLE-ABS-KEY ( "remote consultation" )  OR  TITLE-ABS-KEY ( teletherapy )  OR  TITLE-ABS-KEY ( teletherapy )  OR  TITLE-ABS-KEY ( telecare )  OR  TITLE-ABS-KEY ( tele-care ) ) | [98,510 document results](https://www.scopus.com/search/history/results.uri?origin=searchhistory&shid=4) |
| 2 | ( TITLE-ABS-KEY ( framework* )  OR  TITLE-ABS-KEY ( model* )  OR  TITLE-ABS-KEY ( scale* )  OR  TITLE-ABS-KEY ( dimension* )  OR  TITLE-ABS-KEY ( indicator* )  OR  TITLE-ABS-KEY ( guideline* )  OR  TITLE-ABS-KEY ( metric* )  OR  TITLE-ABS-KEY ( measur* )  OR  TITLE-ABS-KEY ( criteri* )  OR  TITLE-ABS-KEY ( standard* ) ) | [26,767,311 document results](https://www.scopus.com/search/history/results.uri?origin=searchhistory&shid=3) |
| 1 | ( TITLE-ABS-KEY ( information  W/1  quality )  OR  TITLE-ABS-KEY ( data  W/1  quality )  OR  TITLE-ABS-KEY ( information  W/1  valid* )  OR  TITLE-ABS-KEY ( data  W/1  valid* )  OR  TITLE-ABS-KEY ( data  W/1  reliab* )  OR  TITLE-ABS-KEY ( information  W/1  reliab* )  OR  TITLE-ABS-KEY ( data  W/1  accura* )  OR  TITLE-ABS-KEY ( information  W/1  accura* )  OR  TITLE-ABS-KEY ( data  W/1  audit* )  OR  TITLE-ABS-KEY ( information  W/1  audit* ) ) | [235,782 document results](https://www.scopus.com/search/history/results.uri?origin=searchhistory&shid=2) |
