# Supplementary material for: Information Quality Frameworks for Digital Health Technologies: Systematic Review
Source: J Med Internet Res. 2021 May 17;23(5):e23479. doi: 10.2196/23479 (PMC8167621; doi:10.2196/23479)
Supplement: Multimedia Appendix 3 [file jmir_v23i5e23479_app3.docx]

**Multimedia Appendix 3: Included Papers**

| **Study Details** | **Bolt**  **2007** | **McCormack**  **2012** | **Stetson**  **2012** | **Weiskopf**  **2012** | **Almutiry**  **2013** | **Bowen**  **2014** | **Dungey**  **2014** | **Davoudi**  **2015** | **Kahn**  **2016** | **Almutiry**  **2017** |
| --- | --- | --- | --- | --- | --- | --- | --- | --- | --- | --- |
| **Study Methods** | Modification of an existing framework through interviews with developers and users during a pilot of an electronic personal care record. | Ethnographic study – interviews & observation | Modification of existing PDQI through factor analysis, internal consistency assessment | Literature review | Literature Review – DQ dimensions gathering, filtering, mapping & clustering | Not stated | Modification of two existing frameworks and consultation with user group to develop measures | Update of previous practice briefs (1998 and 2012) | Literature review & stakeholders meeting | Mixed – literature review, survey, semi-structured interviews and focus group |
| **Publication type** | Journal article | Conference paper | Journal article | Journal article | Conference paper | Data Quality Evaluation Guide | Conference paper | Practice brief | Journal article | PhD Thesis |
| **Country** | Japan | USA | USA | USA | UK | Canada | UK | USA | USA | UK/ Saudi Arabia |
| **Digital Health Technology** | Web & mobile phone application for telemedicine | CDS | EHR | EHR | Cloud based health information system | Electronic Medical Records | Primary care databases | EHR | EHR | EHR |
| **Name of IQ Framework** | Information quality in home care  coordination services | A descriptive model of factors impacting data quality of CDS | PDQI-9 | DQ assessment in the context of EHR data reuse for research | A framework of DQ in cloud-based health information systems | eHealth Observatory Electronic Medical Records DQ Dimensions | Proposed framework for measuring DQ in primary care research databases | Data Quality Management Model | Harmonized DQ assessment terminology and framework for the secondary use of EHR data | The framework of data quality in EHR |

DQ – Data Quality, PDQI – Physician Documentation Quality Instrument, EHR – Electronic Health Records, CDS – Clinical Decision Support
