# Supplementary material for: Information Quality Frameworks for Digital Health Technologies: Systematic Review
Source: J Med Internet Res. 2021 May 17;23(5):e23479. doi: 10.2196/23479 (PMC8167621; doi:10.2196/23479)
Supplement: Multimedia Appendix 4 [file jmir_v23i5e23479_app4.docx]

**Multimedia Appendix 4: Quality Assessment Result**

| **CASP Checklist** | **Bolt**  **2007** | **McCormack**  **2012** | **Stetson**  **2012** | **Weiskopf**  **2012** | **Almutiry**  **2013** | **Bowen**  **2014** | **Dungey**  **2014** | **Davoudi**  **2015** | **Kahn**  **2016** | **Almutiry**  **2017** |
| --- | --- | --- | --- | --- | --- | --- | --- | --- | --- | --- |
| Was there a clear statement of the aims of the research? | Yes | Yes | Yes | Yes | Yes | NA | Yes | NA | Yes | Yes |
| Is a qualitative methodology appropriate? | Yes | Yes | NA | NA | NA | NA | NA | NA | Yes | Yes |
| Was the research design appropriate to address the aims of the research? | Yes | Yes | Yes | Yes | Yes | NA | Yes | NA | Yes | Yes |
| Was the recruitment strategy appropriate to the aims of the research? | Not clear | Not clear | Not clear | NA | Not clear | NA | Not clear | NA | Not clear | Yes |
| Was the data collected in a way that addressed the research issue? | Not clear | Yes | Yes | Yes | Yes | NA | Not clear | NA | Yes | Yes |
| Has the relationship between researcher and participants been adequately considered? | Not clear | No | NA | NA | NA | NA | No | NA | No | Yes |
| Have ethical issues been taken into consideration? | No | Yes | Yes | NA | No | NA | No | NA | No | Yes |
| Was the data analysis sufficiently rigorous? | No | Yes | Yes | No | No | NA | No | NA | No | Yes |
| Is there a clear statement of findings? | Yes | Yes | Yes | Yes | Yes | NA | Yes | NA | Yes | Yes |
| How valuable is the research? | Yes | Yes | Yes | Yes | Yes | NA | Yes | NA | Yes | Yes |
