# Supplementary material for: Information Quality Frameworks for Digital Health Technologies: Systematic Review
Source: J Med Internet Res. 2021 May 17;23(5):e23479. doi: 10.2196/23479 (PMC8167621; doi:10.2196/23479)
Supplement: Multimedia Appendix 5 [file jmir_v23i5e23479_app5.docx]

**Multimedia Appendix 5: Definition of IQ Dimensions within Existing IQ Frameworks for DHTs**

| **S/N** | **Dimension** | **Extracted Definitions** |
| --- | --- | --- |
| **Information quality in home care coordination services (Bolt 2007)** | | |
| 1 | **Updateability** | Personal data about an individual is likely to need periodic correction or updating by the service users, i.e. care recipients, care givers and care service providers |
| 2 | **Interoperability** | The extent to which an information service can exchange and cooperate in using information from various sources (both adherence to standards and whether systems are able to exchange data with other systems on a technical and policy level) |
| 3 | **Portability** | The ability of the information held by the service to be transferred to another service or service provider |
| **Clinician perspectives on the quality of patient data used for clinical decision support (McCormack 2012)** | | |
| 4 | **Data Accuracy** | The clinicians also complained of inaccurate and unreliable patient data. |
| 5 | **Data reliability** | The clinicians also complained of inaccurate and unreliable patient data. |
| 6 | **Completeness** | Missing or incomplete patient information was the most frequent example clinicians gave of poor data impacting CDS. Data were considered missing when they did not exist in an electronic form |
| 7 | **Data relevance to context** | Many clinicians found that CDS was less effective because it failed to adapt to individual and clinical contexts. |
| 8 | **Accessibility of the data** | Data were considered inaccessible when the information existed, but could not be easily retrieved or used |
| **Physician Documentation Quality Instrument (Stetson 2012)** | | |
| 9 | **Accurate** | The note is true. It is free of incorrect information |
| 10 | **Internal consistent** | No part of the note ignores or contradicts any other part |
| 11 | **Up-to-date** | The note contains the most recent test results and recommendations |
| 12 | **Comprehensible** | The note is clear, without ambiguity or sections that are difficult to understand |
| 13 | **Thorough** | The note is complete and documents all of the issues of importance to the patient |
| 14 | **Useful** | The note is extremely relevant, providing valuable information and/or analysis |
| 15 | **Organised** | The note is well-formed and structured in a way that helps the reader understand the patient’s clinical course |
| 16 | **Succinct** | The note is brief, to the point, and without redundancy |
| 17 | **Synthesized** | The note reflects the author’s understanding of the patient’s status and ability to develop a plan of care |
| **Methods and dimensions of electronic health record data quality assessment: Enabling reuse for clinical research (Weiskopf 2012)** | | |
| 18 | **Completeness** | Is a truth about a patient present in the EHR? |
| 19 | **Correctness** | Is an element that is present in the EHR true |
| 20 | **Concordance** | Is there agreement between elements in the EHR or between the EHR and another data source |
| 21 | **Plausibility** | Does an element in the EHR make sense in light of other knowledge about what that element is measuring |
| 22 | **Currency** | Is an element in the EHR a relevant representation of the patient state at a given point in time? |
| 23 | **Completeness** | Is the data free from significant gaps in coverage that may otherwise limit its ability to represent the true state of affairs? |
| **A framework of DQ in cloud-based health information systems (Almutiry 2013)** | | |
| 24 | **Accuracy** | The extent to which registered data conforms to its actual value |
| 25 | **Completeness** | The state in which information is not missing and is sufficient for the task |
| 26 | **Consistency** | Representation of data values remains the same in multiple data items in multiple locations |
| 27 | **Relevance** | The extent to which information is appropriate and useful for the intended task |
| 28 | **Timeliness** | The state in which data is up to date and its availability is on time |
| 29 | **Usability** | The ease with which data can be accessed, used, updated, understood, maintained and managed |
| 30 | **Provenance** | The source of data, shown and linked to metadata about data |
| 31 | **Interpretability** | The degree to which data can be understood |
| 32 | **Security** | Security prevents personal data from being corrupted and controls access to ensure privacy and confidentiality |
| **EMR Data Quality: Evaluation Guide (Bowen 2014)** | | |
| 33 | **Correctness** | Does the data accurately describe the true of affairs it is intended to represent? |
| 34 | **Concordance** | Is the data in relative agreement with other relevant reputable sources? |
| 35 | **Comprehensibility** | Is the average intended reviewer able to understand the data so as to be able to attempt to infer the author’s intended interpretation of the true state of affairs? |
| 36 | **Informative sufficiency** | Does the body of available data adequately support *an inference* of the true state of affairs by an average intended reviewer irrespective of any objective level of completeness, correctness or consistency? |
| 37 | **Consistency of capture** | Are the desirable data elements consistently recorded? |
| 38 | **Consistency of Form** | Are the data elements consistently captured in the desirable form |
| **A pragmatic approach for measuring data quality in primary care databases (Dungey 2014)** | | |
| 39 | **Accuracy** | Measurement or Recording Error: implausible or incorrect values  Recording accuracy: Coding errors, Recorded date is date event actually happened  Coverage: All events pertaining to a certain condition are recorded. No duplicate records for same event |
| 40 | **Completeness** | Unit non-response: Practice with no data for a period  Item non-response: Practice contributed no data for a certain element or only partial data |
| 41 | **Reliability** | consistency/concordance: information mismatch between various or within the same EHR data source |
| 42 | **Relevance** | Coding specificity: to identify study population  Relevant time intervals: Time intervals adequate for the intended use |
| 43 | **Timeliness** | Practice level timeliness: Practice contributing data to data centre on time  Timeliness of individual records: Practice recording events at time that they happened |
| 44 | **Integrity** | Control of provenance of data |
| 45 | **Validity** | Correct/approved units or specified time intervals  Valid proxy data. |
| **Data quality management model (Davoudi 2015)** | | |
| 46 | **Data Accuracy** | The extent to which the data are free of identifiable errors |
| 47 | **Data consistency** | The extent to which the healthcare data are reliable and the same across **a**pplications |
| 48 | **Data Relevancy** | The extent to which healthcare-related data are useful for the purposes for which they were collected |
| 49 | **Data Timeliness** | Concept of data quality that involves whether the data is up-to-date and available within a useful time frame; timeliness is determined by manner and context in which the data are being used |
| 50 | **Data Currency** | The extent to which data are up-to-date; a datum value is up-to-date if it is current for a specific point in time, and it is outdated if it was current at a preceding time but incorrect at a later time |
| 51 | **Data Accessibility** | Data items that are easily obtainable and legal to access with strong protections and controls built into the process |
| 52 | **Data Comprehensiveness** | All required data items are included—ensures that the entire scope of the data is collected with intentional limitations documented |
| 53 | **Data Definition** | The specific meaning of a healthcare-related data element |
| 54 | **Data Granularity** | The level of detail at which the attributes and values of healthcare data are defined |
| 55 | **Data precision** | Data values should be strictly stated to support the purpose |
| **A Harmonized Data Quality Assessment Terminology and Framework for the Secondary Use of Electronic Health Record Data (Khan 2016)** | | |
| 56 | **Completeness** | focuses on features that describe the frequencies of data attributes present in a data set without reference to data values. |
| 57 | **Conformance** | focuses on DQ features that describe the compliance of the representation of data against internal or external formatting, relational, or computational definitions. |
| 58 | **Plausibility** | focuses on features that describe the believability or truthfulness of data values. |
| **The framework of data quality in EHR (Almutiry 2017)** | | |
| 59 | **Accuracy** | The extent to which registered data conforms to its actual value |
| 60 | **Completeness** | The state in which information is not missing and is sufficient for the task |
| 61 | **Consistency** | Representation of data values remains the same in multiple data items in multiple locations |
| 62 | **Relevance** | The extent to which information is appropriate and useful for the intended task |
| 63 | **Timeliness** | The state in which data is up to date and its availability is on time |
| 64 | **Usability** | The ease with which data can be accessed, used, updated, understood, maintained and managed |
| 65 | **Provenance** | The source of data, shown and linked to metadata about data |
| 66 | **Interpretability** | The degree to which data can be understood. |
| 67 | **Secure access** | Personal data being protected against unauthorised access |
| 69 | **Confidentiality** | The state of information being secret or accessibly restricted under a set of rules that limits the access |
| 70 | **Privacy** | The state of an individual or group being able to seclude themselves or their information |
